# Machine Learning‐Guided Near‐Infrared Laser Processing for Precise Geometry Control in Microneedles with Downstream microLED‐Triggered Photothermal Functionality

**DOI:** 10.1002/smll.74545

**Published:** 2026-07-16

**Authors:** Ismail Eş, Burak M. Gormus, Pinar Ayas, Irfan Ullah, Christopher M. Proctor

**Affiliations:** ^1^ Institute of Biomedical Engineering Old Road Campus Research Building University of Oxford Oxford UK; ^2^ Department of Biomedical Engineering Ankara University Ankara Turkiye; ^3^ Integrated Technologies Application and Research Center (BÜTAM) Ankara University Ankara Türkiye; ^4^ Department of Microsystems Engineering University of Freiburg Freiburg Germany; ^5^ School of Electronics and Computer Science University of Southampton Southampton United Kingdom

**Keywords:** flexible LEDs strip, laser processing, machine learning, metal processing, microneedles, near‐infrared laser, NIR dye

## Abstract

Transdermal drug delivery and sensing provide non‐invasive routes for localized therapy and biomarker detection. Microneedles (MNs) enable pain‐free access to subdermal layers; however, variations in skin thickness and stiffness across anatomical sites demand precise control over MN geometry. This study presents a machine learning‐guided approach for predictive fabrication of MNs using near‐infrared (NIR, 1064 nm) laser processing of duralumin. Laser power, repetition, and computer‐aided design (CAD) dimensions are systematically mapped, and a data‐driven model is trained to predict MN height and tip radius with high accuracy. Using this framework, application‐specific MN geometries are selected prior to fabrication, reducing empirical trial‐and‐error. For example, a target MN height of 524 µm for the female chin is predicted with a 1.24% deviation from experimental measurements. The predictive metal masters are subsequently translated into polymer MNs, demonstrating reliable replication and geometric fidelity. To illustrate functional relevance, polyvinyl alcohol (PVA) MNs incorporating a near‐infrared–responsive dye are fabricated and integrated with a flexible micro–light‐emitting diode (LED) platform, enabling localized photothermal heating. This work establishes an end‐to‐end, data‐driven pipeline linking laser parameters to functional MN devices, offering a scalable strategy for application‐specific MN design across transdermal drug delivery, sensing, and bioelectronic interfaces.

## Graphical abstract

Machine learning‐guided near‐infrared laser processing enables predictive fabrication of microneedles with application‐specific geometries. The optimized metal templates are translated into polymeric microneedles and integrated with a flexible microLED platform. This strategy provides a scalable route toward personalized transdermal drug delivery, sensing, and wearable photothermal therapeutics.

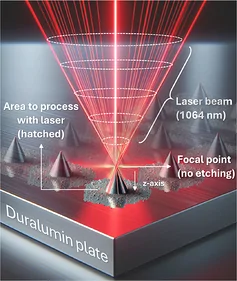

## 1 Introduction

Human skin is an excellent target for delivering drugs, such as messenger RNA (mRNA) vaccines [1], or sensing biomarkers such as glucose [2]. This transdermal area is highly accessible and provides a large surface for interaction, which makes it suitable for both localized and systemic therapeutic applications. Various tools are available for use in this area, including hypodermic needles, which can effectively deliver substances into deeper layers but are inherently invasive, causing pain and potential tissue damage. Conventional transdermal patches, while non‐invasive, are limited to surface‐level application and fail to reach deeper tissues where a broader array of biomarkers resides, restricting their utility in comprehensive sensing and therapeutic delivery [3]. In contrast to hypodermic needles and conventional patches, microneedle (MN) technology plays a vital role in enhancing transdermal drug delivery and sensing by enabling precise and painless access to subdermal layers [4]. This minimally invasive approach facilitates sustained and localized drug release and enhances therapeutic outcomes while reducing potential systemic side effects. Additionally, biosensing through the skin using MNs enables real‐time monitoring and provides continuous insights into ongoing biological processes without the need for invasive blood sampling.

MNs are micron‐sized needle arrays organized on a patch, with heights ranging from tens to hundreds of micrometers, and in some cases, slightly exceeding 1 mm [5]. MNs need to pierce the stratum corneum, the outermost layer of the epidermis, and reach the deeper layers of the epidermis or dermal layer to be effective in transdermal applications. The skin, recognized as the largest organ in the human body, exhibits significant variation in the thickness of both the epidermis and dermis layers depending on the specific area. For instance, the epidermis on the eyelids is exceptionally thin, measuring approximately 0.05 mm, while on the palms and soles, it can be up to 1.5 mm thick [6]. In contrast, the dermis shows even greater variation, with measurements ranging from about 2.1 mm on the dorsum of the hand in women to nearly 5.9 mm in the breast area of men [7]. Additionally, certain areas of human skin exhibit varying levels of stiffness (also referred to as skin elasticity), requiring MNs to adapt accordingly [8]. Pathological conditions, for instance, Ehlers‐Danlos syndrome can further alter skin stiffness, affecting MN design and application strategies [9]. In some cases, they need to be sharper to effectively pierce the outermost layer, while in others, they require a more balanced aspect ratio—the ratio between height and base diameter—to avoid breakage and withstand mechanical stress during movement in those areas. Therefore, the geometrical features of MNs, such as height, aspect ratio, tip diameter, and tip angle, should be carefully designed and tailored to suit the specific application area of the skin.

MNs are typically used in four main types: solid MNs, hollow MNs, coated MNs, and dissolving MNs. Solid MNs are simple, needle‐like structures used to pierce the skin and create microchannels [10]; hollow MNs have a central lumen allowing for direct delivery of drugs [11]; coated MNs are solid structures coated with a drug or sensing material that dissolves or interacts with the tissue upon insertion [12]; and dissolving MNs are made of biodegradable materials that release drugs as they dissolve within the skin [13]. Solid MNs represent the simplest form, and their fabrication is critical as this structure serves as a foundation for developing other MN types. They are fabricated using a variety of materials but are generally made from more rigid substances, such as silicon [4], metals [14], or polymers like polymethyl methacrylate (PMMA) [15]. Solid MNs can be produced using various techniques, including 3D printing [16], photolithograph [4], electrical discharge machining (EDM) [17], and mechanical cutting tools such as dicing saws [18]. While these techniques have been shown to be effective, they still have certain limitations in achieving MNs with the desired height. Moreover, some methods, such as photolithography and EDM, are associated with high operational and equipment costs. Therefore, faster, simpler, and considerably more cost‐effective techniques are preferable. To this end, laser micromachining has become an increasingly important fabrication route, particularly in bioelectronics, to offer precise control over ablation and material modification across metals and polymers [19]. For instance, femtosecond pulsed lasers have been used to microfabricate thin‐film and microscale architectures for biomedical devices [20]. As a more specialized implementation of this approach, near‐infrared lasers have demonstrated high effectiveness in processing metals to create microneedles with the desired geometric properties.

With such laser systems, it is feasible to create MN arrays on various metals, ranging from Duralumin [21, 22] and brass to stainless steel or titanium. Recent work using dynamically autofocused pulsed‐laser systems has shown that these tools can rapidly sculpt complex 3D microstructures with high spatial fidelity [23]. Yet, such processing with NIR lasers requires intensive optimization for each type of metal. For example, the outcome of MN geometry varies significantly depending on factors such as laser power, scan speed, laser etching repetition, and CAD design dimensions. Obtaining such information can be utilized in machine learning models to provide insights into the precise parameters needed to achieve MNs with specific properties, such as MNs with enhanced sharpness, a smaller aspect ratio, and an approximate target height. Training these models on available laser processing datasets, including height maps encoded as heat maps for each MN array and geometrical descriptors extracted from 3D scanning microscopy, enables machine learning algorithms to learn feature representations that capture underlying patterns and parameter dependencies. By leveraging predictive modeling and feature‐space analysis, these models can optimize fabrication parameters, reduce the reliance on heuristic‐based trial‐and‐error approaches, and improve reproducibility in MN manufacturing [24]. Machine learning algorithms, including regression models, support vector machines (SVM), and neural networks, can effectively learn complex mappings between laser processing parameters and MN geometry [25]. This predictive capability enables faster refinement of MN designs and facilitates rapid customization for specific therapeutic or sensing applications.

To our knowledge, this is the first study to integrate ML with NIR laser ablation to accurately predict and control MN geometry. While previous machine learning‐assisted laser studies have primarily focused on general process optimization, signal analysis, or manufacturing monitoring, the present work specifically establishes a predictive fabrication workflow for transdermal MN engineering and downstream wearable photothermal device integration. Unlike prior laser‐based MN fabrication works, our study systematically maps the multidimensional parameter space (laser power, repetition, and CAD size) and establishes a predictive model capable of selecting fabrication conditions for a desired MN height. In this study, we used a feedforward artificial neural network (ANN) to optimize near‐infrared laser (1064 nm) processing parameters for fabricating Duralumin MNs with precise geometries for transdermal applications. The ANN architecture consisted of three input neurons (laser power, CAD size, and laser repetition), four hidden layers (8, 16, 32, and 8 neurons), and an output layer that predicted either MN height or tip radius. The model was trained using the Adam optimizer with a mean squared error (MSE) loss function. It utilized early stopping and dataset splits of 60%–20%–20% for height prediction and 90%–5%–5% for tip radius estimation. By adjusting CAD design dimensions, laser power, and repetition counts, we achieved controlled variations in MN height, base diameter, aspect ratio, tip angle, and tip radius to match specific skin characteristics. To assess the model's predictive accuracy, we compared the predicted MN height with experimentally manufactured MNs for various skin sites, including the forearm, dorsum of the hand, abdomen, dorsum of the foot, breast, central forehead, and chin. The model's performance was then quantified using mean absolute error (MAE), root mean squared error (RMSE), and coefficient of determination (R^2^). Our machine learning‐assisted approach enabled efficient, repeatable fabrication of MNs, demonstrating a reliable method for producing MNs with the precise geometries needed for targeted therapeutic and sensing applications. In addition to computational predictions and experimental validation, we extended our workflow to demonstrate feasible application of the MNs. Recent studies have also explored photothermally responsive MN systems incorporating functional nanomaterials such as MXene for controllable therapeutic release and wound healing applications, further highlighting the growing interest in integrating photothermal functionality into MN platforms [26]. We fabricated a PVA MN array hybridized with a polycaprolactone (PCL) layer incorporating a robust photothermal dye. Due to their ability to penetrate the skin, MNs are very promising for drug delivery and wound healing. For instance, recent studies have also demonstrated multifunctional hydrogel‐based MN systems incorporating antibacterial and proangiogenic functionalities for enhanced wound healing applications [27]. Particularly, there are approaches showing efficient treatment using heat where microheaters have been used as heat sources via electrical stimulation or more effectively, heat can be obtained by the excitation with NIR laser source. However, handling laser sources can be challenging in terms of patients as non‐expert users and require extra care. Recent studies have also demonstrated the potential of photothermal‐responsive MN systems for controllable therapeutic delivery and wound healing applications using near‐infrared activation, including porous hollow MN architectures capable of heat‐triggered release [28].

To exploit NIR light in a minimized and integrated way, we designed a MN system with a photothermal therapeutic function that can be stimulated in a wearable and smart platform. We designed a flexible strip made from polyimide copper‐coated film and high‐power mini NIR LEDs, and an LED driver chip was embedded to it. MN array showed a significant and controllable heat generation capability upon stimulation with the battery‐powered strip, which shows its potential in localized photothermal therapy. By leveraging the predictive power of our model, researchers and practitioners can enhance the fabrication process, enabling the efficient generation of MNs with tailored geometries to meet specific application requirements. This work introduces an end‐to‐end pipeline that links ML‐optimized metal masters with polymer MN fabrication, eventually reducing fabrication variability and creating a consistent route from laser parameters to final geometry. Collectively, these advances establish a new paradigm for data‐driven MN manufacturing, enabling predictive, application‐specific fabrication that has not previously been achieved with NIR laser‐processed metal templates.

## 2 Results and Discussion

**FIGURE 1:**
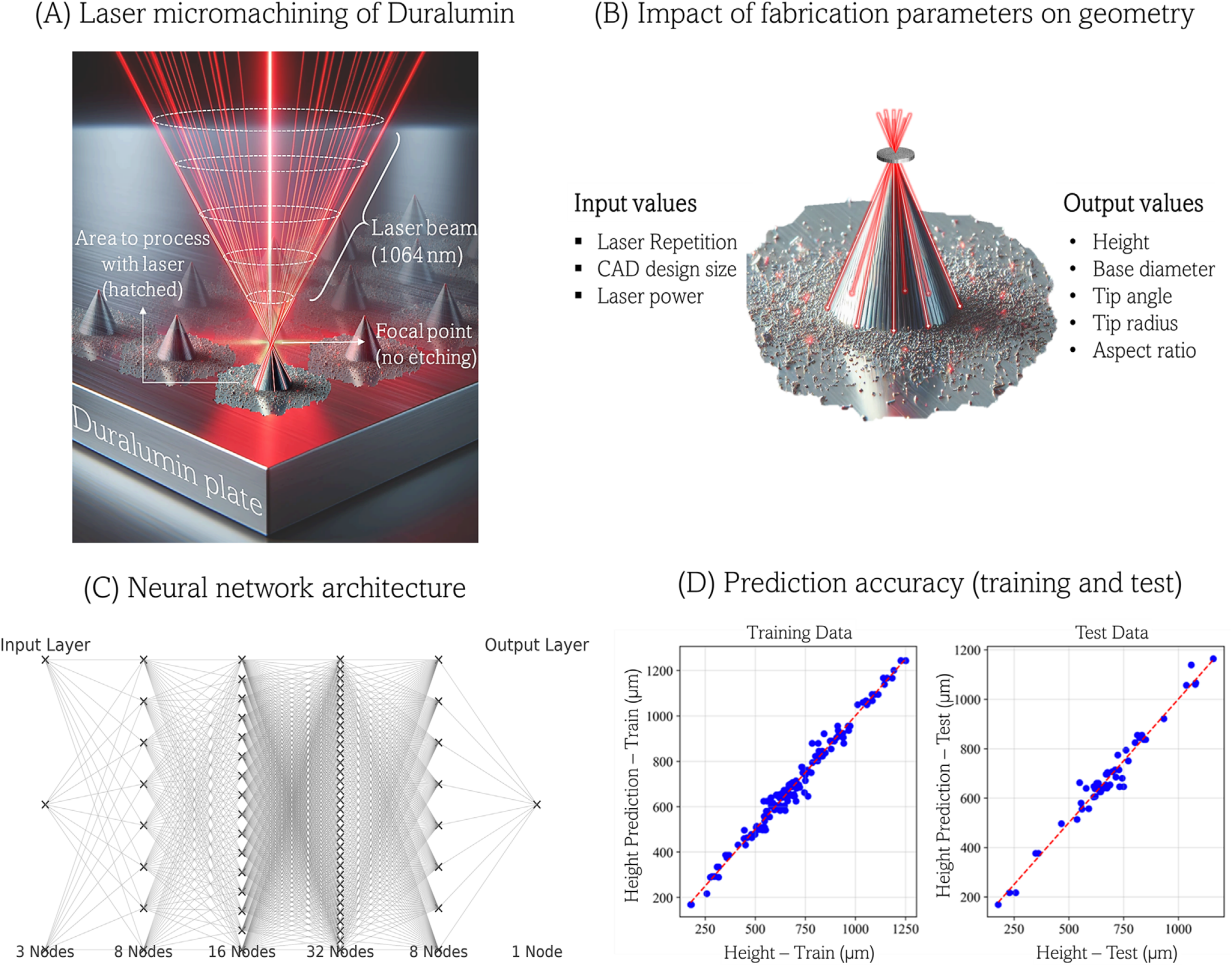
Illustration of the laser processing principle and fabrication steps for producing solid microneedles (MNs). **(A)** Schematic illustration of the near‐infrared (1064 nm) laser micromachining process used to generate MN templates on a duralumin substrate. **(B)** Machine‐learning framework relating laser input parameters to MN geometric outputs. **(C)** Neural network architecture implemented to predict MN height based on selected laser settings. **(D)** Validation of the machine‐learning model using training and test datasets to confirm accurate prediction of MN height prior to fabrication.

MN formation is highly dependent on the selection of laser processing parameters, with each parameter influencing the resulting MN features. In this study, we investigated the effects of CAD‐generated design, laser power, and repetition on MN characteristics, including height, base diameter, aspect ratio, tip angle, and tip radius. The data obtained from these experiments were then introduced into a machine learning model to analyze how each parameter impacts MN formation, which helps us understand the interplay between design and processing conditions in achieving optimal MN structures. This integrated workflow—from laser ablation to predictive modeling and final MN fabrication—is summarized in Figure 1. This ML‐selected geometry was then directly transferred into the molding workflow, ensuring that all downstream polymer MNs—including those used for photothermal testing—inherited the optimized dimensions.

### 2.1 Parameter Screening for Efficient Laser Ablation of MNs

#### 2.1.1 Metal Selection

The selection of metal plays a crucial role in MN fabrication using a 1064 nm laser, as different metals exhibit varying responses to the laser beam due to their unique mechanical and thermal properties [29]. In this study, duralumin was found to be more suitable for MN fabrication, achieving higher MN heights with optimized laser power and repetition settings. The superior processability of these metals can be attributed to their lower thermal conductivity and higher energy absorption capacity, which facilitate efficient ablation and reduce heat dissipation away from the laser interaction zone [30]. These properties allow the laser to concentrate energy effectively on the surface, enabling precise etching and the formation of well‐defined MNs. We found that metals like duralumin are more suitable for processing with a 1064 nm laser due to their lower melting points, moderate thermal conductivity, and higher energy absorption. These properties allowed us to achieve efficient ablation and precise MN formation, making them preferable choices for laser‐based fabrication methods.

#### 2.1.2 Laser Power

In our study, we selected laser power settings of 20%, 25%, 30%, 35%, and 40% to process the Duralumin plate. This range was chosen based on preliminary observations indicating that laser powers below 20% resulted in insufficient energy delivery, merely etching the surface without achieving the desired MN geometry at any CAD design parameter or repetition values. Conversely, laser powers exceeding 40% led to surface deformation and increased roughness on the MN array. Such elevated roughness can adversely affect the mechanical stability and penetration efficacy of MNs and reduce their reliability in transdermal applications. Therefore, maintaining laser power within the 20% to 40% range was essential to balance effective material processing while preserving MN integrity.

#### 2.1.3 Laser Repetition

We tested laser repetition settings ranging from 50 to 400 repetitions in steps of 50 to optimize the MN fabrication process. At 50 and 100 repetitions, the energy delivered was not sufficient to form MN tips, resulting in irregularly shaped pillar‐like structures, as shown in Figure S1. Repetition values exceeding 400 led to excessive etching through the Duralumin plate, producing structures with heights surpassing 1000 µm, which deviated from the MN features reported in the literature. Additionally, at higher repetition values, the laser continued to interact with the already formed MNs, progressively ablating material and reducing their height with each additional repetition step, further deviating the geometry from the desired specifications. Beyond a certain repetition threshold, the increased depth of focus caused the laser beam intensity to weaken at the lower regions of the plate, ultimately failing to etch material effectively in these areas.

#### 2.1.4 CAD‐Generated Design Dimensions

To identify the optimal CAD design, we tested both circular and square shapes in AutoDESK AutoCAD, with diameters or side lengths ranging from 50 µm to 150 µm in 25 µm increments. Initial tests showed that the 25 µm CAD design failed to form MN tips, as the structure size was too small relative to the laser precision, causing the beam to pass over the intended features and produce a flat surface instead. Conversely, the 125 µm CAD design was too large, necessitating additional laser repetition steps, which significantly increased processing time. As a result, we focused on CAD dimensions of 50, 75, and 100 µm to balance efficient laser processing with successful MN formation.

Although additional parameters such as scanning speed, hatch spacing, pulse frequency, energy density, and spot size can influence laser‐material interactions, these variables were intentionally fixed during this study to maintain a manageable experimental design space and establish an initial machine learning framework focused on the dominant fabrication parameters. The laser spot size remained constant due to the fixed optical configuration of the 1064 nm laser system used in this work. Nevertheless, spot size should be carefully considered for different 1064 nm laser systems employing different focusing optics, focal lengths, beam quality, or pulse characteristics, as these factors can substantially alter the laser‐material interaction and resulting MN geometry. Similarly, scanning speed and pulse frequency were maintained constant because both parameters are strongly coupled with effective energy delivery during ablation and would substantially increase the number of experimental combinations required for model training. Preliminary experiments further indicated that hatch spacing mainly affected the density and arrangement of MNs within the array rather than the geometry of individual MNs, provided that excessive overlap between adjacent laser tracks was avoided. Moreover, although variations in scanning speed and pulse frequency influenced local ablation behavior and surface morphology, the final achievable MN height approached a similar threshold due to the fixed focal position of the laser beam. As the etched depth increased, the laser gradually moved away from the optimal focal plane, reducing the effective energy delivered to deeper regions and consequently limiting further vertical growth of the MN structures. Therefore, laser power and repetition remained the dominant parameters governing the final MN geometry under the selected processing conditions.

### 2.2 MN Formation Using Laser Processing

In this study, we used a 1064 nm laser to precisely ablate the surface of duralumin plate for MN formation. To achieve consistent results, we maintained a fixed *z*‐axis alignment for both the sample and the laser beam. As ablation progressed, the laser beam's interaction area naturally extended beyond the initial focal point due to the increasing depth of the etched region, forming a depth‐of‐focus zone [21]. We observed that the laser's consistent targeting and gradual material ablation led to precise control over the MN geometry throughout the fabrication process. By maintaining a stable focal alignment, the laser effectively shaped the MNs with high accuracy and produced structures with desired dimensions. We exploited this phenomenon to create MNs with customized geometries.

As previously aforementioned, we tested laser powers between 20% and 40%, as powers below 20% did not provide sufficient energy to create an effective etching area, while powers above 40% risked overheating the material and affecting the surface structure. To avoid complicating the machine learning algorithm and increasing the required sample size, we maintained the scan speed at 500 mm/s and the pulse frequency at 100 mHz, where pulse frequency refers to the number of laser pulses emitted per second, determining the energy distribution and precision of the laser‐material interaction. These parameters were kept constant because they are directly related to laser power and could introduce additional variability into the algorithm. During the initial screening stage, additional CAD design sizes (25 µm, 100 µm, and 125 µm) were also investigated. Considering all CAD design levels, laser power levels, laser repetition levels, and triplicate fabrication, the initial experimental matrix consisted of 600 fabricated MN samples. For each sample, five geometrical parameters (height, base diameter, aspect ratio, tip angle, and tip radius) were extracted through image analysis, resulting in approximately 3000 measured geometrical values. However, some CAD design conditions did not produce well‐defined MN structures and were therefore excluded from the final statistical and machine learning analysis. The final dataset used for the machine learning model consisted of 360 fabricated MN samples.

#### 2.2.1 MN Height

**FIGURE 2:**
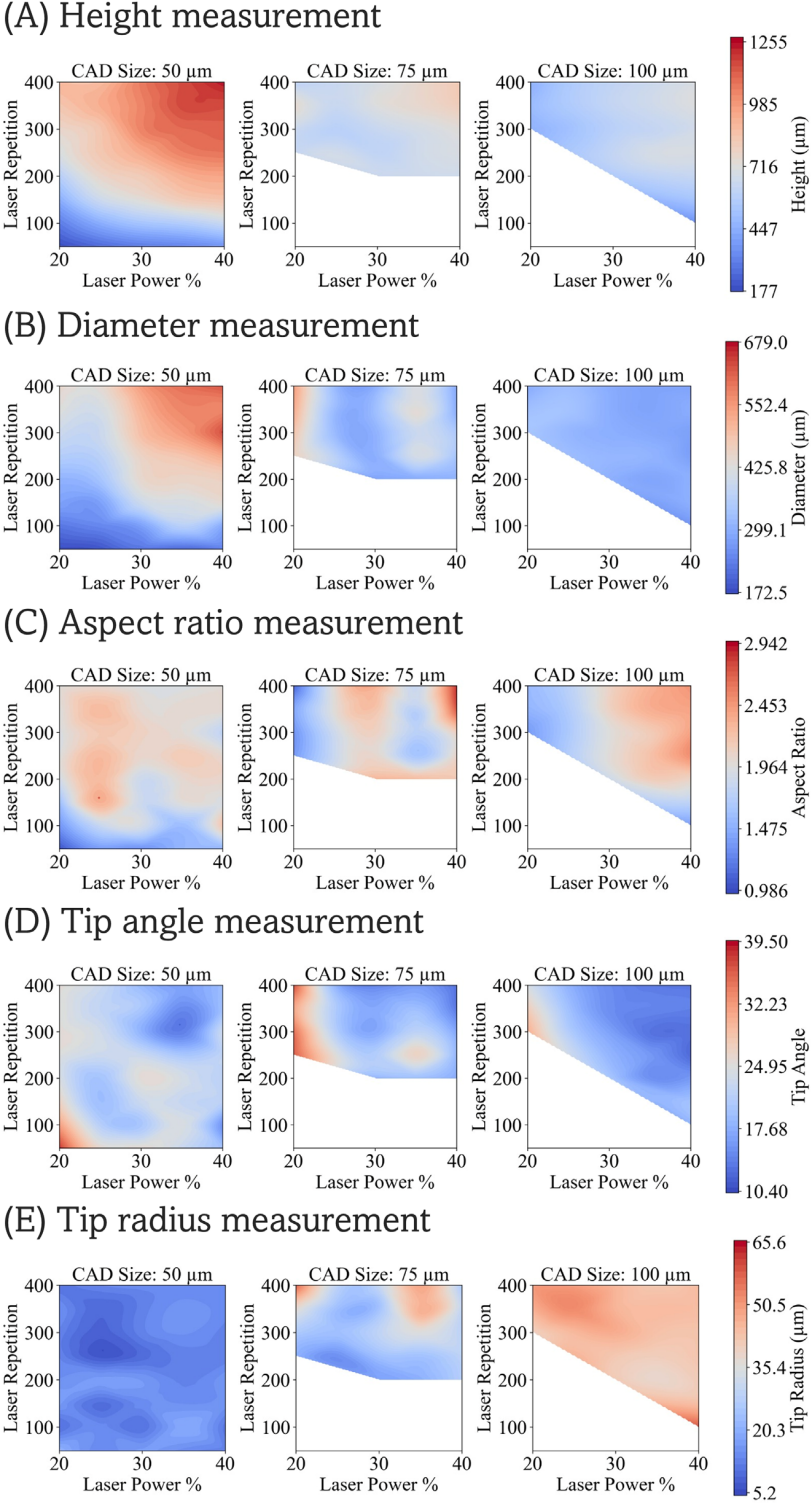
Heatmap representation of the experimental MN fabrication results for different CAD sizes (50, 75, and 100 µm) as a function of laser power (%) and laser repetition. (A) Height measurement, (B) Diameter measurement, (C) Aspect ratio measurement, (D) Tip angle measurement, (E) Tip radius measurement. White regions indicate parameter sets that resulted in low‐quality MNs and were excluded from further analysis. Statistical significance of the investigated parameters was evaluated using ANOVA analysis. All main factors and interaction terms demonstrated statistically significant effects on microneedle geometry (*p* < 0.001).

MN height is a critical parameter, as different parts of the skin exhibit varying thicknesses, requiring tailored designs for specific applications. Ensuring optimal MN height is essential to achieve effective penetration while minimizing discomfort and ensuring accurate drug delivery or sensing. The effect of laser parameters on MN height, base diameter, aspect ratio, tip angle, and tip radius, which are the most critical parameters in defining MN efficiency, is shown in Figure 2. To further clarify these variations and highlight statistical differences, Figure S2 in the supplementary material presents the same data in bar plots with a detailed comparative analysis of the fabrication process.

According to our findings, the laser power significantly affects the height of MNs fabricated using a 1064 nm laser. As the laser power increases, the height of the MNs consistently grows, which demonstrates a clear correlation between energy input and material ablation. This trend is evident across different CAD sizes (50 µm, 75 µm, and 100 µm), with the MN height reaching its peak at higher laser power and laser repetition values. For each CAD design (50 µm, 75 µm, and 100 µm), an increase in laser repetition at the same laser power consistently resulted in a significant increase in MN height. For example, at 30% laser power with a CAD size of 50 µm, the MN height increased from approximately 200 µm at 50 repetitions to over 1000 µm at 400 repetitions. Similarly, for a CAD size of 75 µm, the height grew from around 300 µm at 50 repetitions to nearly 1200 µm at 400 repetitions. The trend is valid across all CAD designs, which shows that higher laser repetitions enhance the ablation process, thereby increasing the MN height.

The effect of laser repetition on MN height shows a clear initial increase, particularly between lower repetition values. For example, when laser repetition increased from 50 to 100 and then from 100 to 150, there was a significant rise in height for all CAD designs. However, after a certain number of repetitions—typically between 300 and 350—especially at higher laser powers such as 30%, 35%, and 40%, the rate of height increase slows down. This behavior can be attributed to the laser beam reaching its energy threshold for effective ablation. As the MN grows taller, the *z*‐axis position of the laser remains fixed, which causes the focal point of the laser to move further away from the plate surface. Consequently, the energy delivered to the surface diminishes as the focal point diverges, limiting the ablation efficiency. This phenomenon results in a reduced height increment at higher repetition values, eventually approaching a threshold where further repetition does not significantly enhance the MN height.

#### 2.2.2 MN Base Diameter

The base diameter of MNs was observed to increase significantly with laser power. At a laser power of 20%, the base diameter ranged from approximately 150 µm (at 50 repetitions) to around 350 µm (at 400 repetitions). With increasing laser power to 40%, the base diameter expanded further, reaching values as high as 600 µm under similar repetition conditions. The larger base diameters at higher power levels can be attributed to the increased energy delivered to the surface, which broadens the laser interaction zone.

Laser repetition had a marked impact on the base diameter, as the diameter increased consistently with repetitions rising from 50 to 400. For example, at 30% laser power and a CAD size of 50 µm, the base diameter grew from approximately 250 µm at 50 repetitions to about 500 µm at 400 repetitions. This trend is explained by the cumulative ablation effect of successive laser pulses. As more material is removed with each repetition, the z‐axis depth increases, leading to a greater distance between the laser's focal point and the deepest point of the etched region. This results in a wider laser beam interaction zone due to the Gaussian nature of the beam, which spreads as the focus point deviates from the surface (Figure 1A). Consequently, the ablation area expands laterally, causing the base diameter to widen.

Quantitative data further reveals the influence of CAD size on base diameter under varying repetitions. At 30% power and 400 repetitions, the base diameter for a CAD size of 50 µm was approximately 500 µm, while for 75 µm and 100 µm CAD sizes, the diameters reached around 600 µm and 700 µm, respectively. This proportional increase indicates that larger CAD designs inherently lead to broader bases, as the initial laser footprint is wider. The effect of laser repetition amplifies this trend, particularly for larger CAD sizes, by accentuating the lateral spread of the ablation zone with increasing depth.

The base diameter of MNs, influenced by laser repetition and CAD size, plays a crucial role in their application. Larger base diameters, achieved with higher repetitions and larger CAD sizes, enhance mechanical stability, making them ideal for transdermal drug delivery or hollow MNs requiring greater structural robustness. For hollow MN systems, lumen diameter additionally represents an important geometrical parameter because it directly influences fluid transport resistance, delivery efficiency, and mechanical stability of the MN structure. Therefore, future machine learning‐assisted optimization studies involving hollow MN fabrication may benefit from incorporating lumen diameter together with MN height, base diameter, tip geometry, and interspacing distance as predictive output variables. In contrast, smaller base diameters, associated with fewer repetitions or smaller CAD sizes, are better suited for minimally invasive applications, such as cosmetic treatments or high‐density MN arrays for vaccine delivery, where reduced trauma and closer needle spacing are essential.

In addition to individual MN geometry, the interspacing distance between adjacent MNs is another important design parameter influencing transdermal performance. Previous studies demonstrated that smaller MN spacing can significantly increase local skin strain and mechanical interaction between neighboring MNs, potentially reducing penetration efficiency due to the “bed of nails” effect. It has been reported that MN spacings below approximately 2–3 mm significantly increased skin strain and needle reaction force during insertion simulations [31]. Similarly, MN arrays were investigated with interspacing distances ranging from 0.156 to 1.75 mm to evaluate neighboring MN interactions during insertion [32]. Conversely, excessively sparse MN arrangements may reduce effective tissue contact area and overall drug delivery efficiency.

From a fabrication perspective, the center‐to‐center interspacing distance was maintained at 1.2 mm throughout this study, which was sufficiently larger than the minimum spacing required to avoid interactions between adjacent MNs during laser processing. Additional fabrication trials performed with interspacing distances as low as 0.5 mm did not reveal observable differences in MN formation or geometry. This indicates that neighboring MN fabrication did not influence the resulting structures under the processing conditions employed. Therefore, the developed machine learning framework captures the relationship between laser processing parameters and individual MN geometry without interference from neighboring structures. Since fabrication‐related interactions were not observed even at interspacing distances substantially smaller than those used in this study, the obtained relationships between laser parameters and MN geometry can reasonably be extended to MN arrays fabricated with larger interspacing distances, where neighboring MN interactions during fabrication are expected to be even less significant.

In the present study, interspacing distance was intentionally maintained constant to isolate the effects of laser processing parameters on individual MN geometry. Nevertheless, integrating array‐level design parameters such as MN density and interspacing distance into future machine learning frameworks could further improve application‐specific optimization of MN arrays for different skin types and therapeutic applications.

#### 2.2.3 Tip Radius

The tip radius of the MNs decreases with increasing laser repetitions up to a certain point, but the trend stabilizes at higher repetition rates. At 20% laser power and a CAD size of 50 µm, the tip radius reduced from approximately 25 µm at 50 repetitions to around 10–15 µm by 250–400 repetitions. This reduction is due to repeated ablation refining the tip, removing excess material, and creating sharper structures. However, at higher repetitions, the tip radius stabilizes due to a balance between material removal and heat‐induced redeposition.

Increasing the laser power tends to yield smaller tip radii, as higher energy density focuses the ablation more precisely at the tip. For instance, at 40% power with a CAD size of 75 µm, the tip radius reduced to about 5–10 µm at 400 repetitions, compared to 15–20 µm at lower powers like 20%. This demonstrates the enhanced ability of higher powers to sculpt sharper MNs. However, excessive power could risk material deformation at the tip due to localized overheating.

The CAD size also influences the tip radius, with smaller CAD sizes producing finer tips. For example, at 30% laser power, the tip radius for a CAD size of 50 µm remained consistently smaller (10–15 µm) compared to 75 µm and 100 µm CADs, where the tip radii were slightly larger, ranging from 15 to 20 µm under similar conditions. This is because smaller CAD sizes confine the initial laser interaction area, promoting sharper geometry during the etching process.

In our study, we excluded data from specific CAD designs and laser parameter combinations as shown in Figure 2 due to the formation of nonuniform tip radii, which made it impossible to measure the tip radius and other critical MN features. For the 75 µm CAD design, the excluded parameters included laser power of 20 W with repetitions of 50, 100, 150, and 200; laser power of 25 W with repetitions of 50, 100, 150, and 200; laser power of 30 W with repetitions of 50, 100, and 150; laser power of 35 W with repetitions of 50, 100, and 150; and laser power of 40 W with repetitions of 50, 100, and 150. Similarly, for the 100 µm CAD design, we excluded laser power of 20 W with repetitions of 50, 100, 150, 200, and 250; laser power of 25 W with repetitions of 50, 100, 150, and 200; laser power of 30 W with repetitions of 50, 100, and 150; laser power of 35 W with repetitions of 50, 100, and 150; and laser power of 50 W with a repetition of 50. These parameter combinations were excluded as they resulted in inconsistent tip geometries, rendering the data unsuitable for further analysis.

The tip radius of MNs is a critical parameter in determining their efficiency and functionality across various applications. A smaller tip radius is essential for minimally invasive applications, as it reduces the insertion force required to penetrate the skin or other biological barriers, enhancing user comfort and ensuring consistent delivery or sampling. Additionally, sharp tips improve precision in targeted drug delivery or biosensing, minimizing tissue damage and maximizing therapeutic or diagnostic efficiency. In general, smaller tip radii are preferred because sharper MN tips reduce the insertion force required to penetrate the skin or other biological barriers, thereby improving penetration efficiency and minimizing tissue trauma. Additionally, sharp tips can enhance precision in targeted drug delivery and biosensing applications. Nevertheless, tip sharpness should also be considered together with the mechanical robustness of the MN structure and the intended application site. Extremely sharp tips, particularly in polymeric MN systems, may become more susceptible to bending or fracture under mechanically dynamic conditions, such as applications on joints or highly curved skin regions exposed to repeated movement and shear stress. Therefore, practical MN design often involves balancing penetration efficiency with structural stability depending on the target application.

#### 2.2.4 MN Surface Characteristics

**FIGURE 3:**
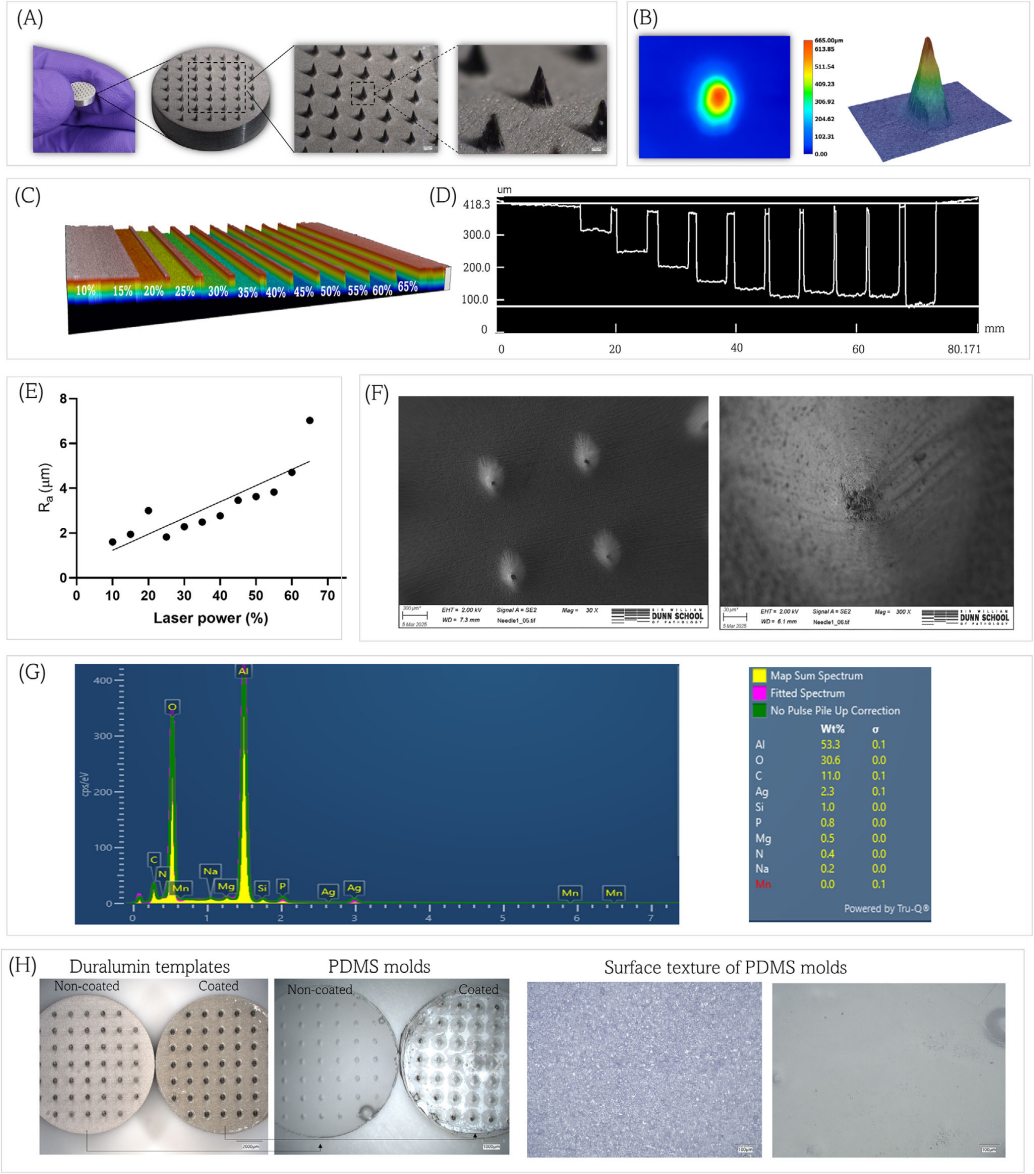
Characterization of MNs fabricated on an aluminum substrate. (A) Fabrication of MNs, including a photograph of the fabricated MN array held with tweezers, a 3D‐printed mold containing MN structures, an optical microscopy image of the MN array, and a close‐up optical microscopy image showing individual MNs. (B) A heat map representing the height distribution of a single MN obtained from a digital microscope. (C) Surface profilometry data illustrating the MN height variation across different laser processing parameters. (D) Depth profile measurement confirming the MN height across the substrate. (E) Roughness values of the aluminum surface after laser processing, showing variations between 10% and 65% processing intensity. (F) Scanning electron microscopy (SEM) images taken from the top view of the MN array, along with a high‐magnification SEM image demonstrating the tip uniformity of a single MN. (G) Energy‐dispersive X‐ray spectroscopy (EDS) spectrum confirming the elemental composition of the MNs, accompanied by an EDS quantification table displaying the weight percentages of detected elements. (H) Optical images comparing non‐coated and coated MN master templates and the corresponding polydimethylsiloxane (PDMS) molds obtained through soft lithography, as well as surface texture analysis of PDMS molds replicated from non‐coated and coated master templates.

The structural and surface properties of the fabricated MNs were systematically analyzed to assess how laser processing influenced their morphology and material characteristics. As shown in Figure 3A–G, we examined MN height uniformity, surface topography, roughness evolution with laser power, tip integrity, and elemental composition of the ablated regions. Based on these results, we first focused on quantifying the surface roughness of duralumin under different laser power levels, as roughness plays a critical role in MN mechanical stability and molding performance.

#### 2.2.5 Roughness

Examining the surface roughness of duralumin after exposure to varying laser power levels helps to understand the material's behavior and changes during near‐infrared laser etching (Table 1). As observed in the roughness measurements, increasing laser power generally leads to higher Rz, Ra, and Rq values, indicating greater material ablation and surface modification. As observed in the roughness measurements, increasing laser power generally leads to higher Rz, Ra, and Rq values, which is also reflected in the 3D surface topography for each power level (Figure S3). The roughness starts at 1.601 µm when the laser power is set to 10%, indicating minimal surface modification. As the laser power increases to 15% (Ra = 1.941 µm) and further to 20% (Ra = 3.001 µm), a noticeable increase in roughness occurs, suggesting enhanced material removal and microstructural changes due to localized heating. At higher laser powers, such as 25% (Ra = 1.824 µm) and 30% (Ra = 2.282 µm), the material undergoes a complex interaction with the laser, leading to a decrease in Ra compared to 20%, possibly due to the onset of material melting and resolidification effects. However, as laser power continues to rise to 35% (Ra = 2.491 µm) and 40% (Ra = 2.770 µm), roughness increases again, consistent with a more aggressive etching process that removes material more unevenly. The highest roughness values are observed at 50% (Ra = 4.709 µm) and 55% (Ra = 7.032 µm), where extensive surface deformation and deep microstructures are formed, affecting material integrity. Earlier research on aluminum processing with various laser sources resulted in diverse surface characteristics [33, 34, 35].

**TABLE 1:** Surface roughness parameters (Rz, Ra, and Rq) of duralumin after etching with different laser power of a near‐infrared laser. Each value was obtained from samples processed under varying laser power settings, assessing the surface properties in response to the etching process.

Laser power (%)	Ra (µm)	Rz (µm)	Rq (µm)
10%	1.601	15.2	2.002
15%	1.941	18.455	2.399
20%	3.001	31.806	3.877
25%	1.824	26.587	2.303
30%	2.282	22.343	2.834
35%	2.491	29.496	3.15
40%	2.77	38.976	3.686
45%	3.465	53.657	4.649
50%	3.629	31.927	4.526
55%	3.833	40.433	4.914
60%	4.709	47.495	6.081
65%	7.032	67.996	8.792

Ra: Arithmetic mean roughness, representing the average deviation of the surface profile from the mean line, commonly used to assess overall surface smoothness. Rz: Maximum height of the roughness profile, determined from the vertical distance between the highest profile peak and the deepest profile valley within the relevant sampling length or profile section and averaged according to the applied measurement standard. Rq: Root mean square (RMS) roughness, measuring the square root of the mean squared deviations from the mean line, giving higher weight to larger peaks and valleys compared to Ra.

For MN applications, surface roughness plays an important role in fabrication quality, molding behavior, and mechanical integrity of the fabricated structures [3]. In the present study, increasing laser power altered the surface morphology of the duralumin substrate, which directly influenced the consistency of the laser‐processed MN structures. Particularly at higher laser powers, excessive roughness was associated with more aggressive ablation behavior and local surface deformation, potentially affecting the structural uniformity and mechanical robustness of the MNs. Additionally, surface roughness influenced the PDMS molding and peel‐off process, where rougher untreated templates showed stronger adhesion to PDMS during soft lithography replication. Therefore, maintaining an appropriate laser power range is important to balance efficient material processing, structural consistency, and reproducible mold fabrication.

#### 2.2.6 PDMS Peel‐Off Validation From Laser‐Ablated Mold

In this study, we prepared PDMS and used the laser parameters predicted by our machine learning model to produce MNs with a predicted height of 711 µm, targeting the plantar foot as a model. Initial attempts to peel off PDMS from the laser‐processed Duralumin template were challenging due to the rough surface, which led to strong adhesion and difficulties in mold removal. This behavior was consistent with the increased surface roughness values quantified in Table 1 for laser‐processed duralumin surfaces, confirming that laser‐induced roughness directly influenced PDMS‐template adhesion during molding. To address this, the surface of the Duralumin template was treated by cleaning with isopropanol and sonicating for 15 min, followed by a surfactant‐based approach. A 2% MICRO‐90 Alkaline Cleaner solution was poured onto a circular metal template, protected with Kapton tape on the sides, and heated on a hot plate at 100°C to deposit a thin surfactant layer on the MN surface. Repeating this process ensured consistent coverage, which significantly reduced adhesion, allowing successful peeling of PDMS despite the template's rough surface. To document this process, Figure 3H compares PDMS molds obtained from untreated and surfactant‐treated masters, illustrating the improved peel‐off performance after surfactant deposition. Importantly, the thickness of the PDMS layer played a crucial role, as thinner layers tended to break during peeling, while a thickness of approximately 1 cm facilitated easy removal and preserved PDMS mold integrity. After peeling off the PDMS, we examined the structures under a microscope and confirmed that their height matched the master metal mold, with no observable differences (Figure S4). The metal MN exhibited a height of 673.49 µm, while the replicated PDMS structure showed a height of 673.05 µm. Therefore, the replication fidelity, which represents how accurately the PDMS reproduced the original metal MN geometry, was calculated to be approximately 99.93%, while the percentage deviation between the master and replicated structure was only 0.065%. The resulting PDMS molds demonstrated reusability and could be effectively cleaned or autoclaved for use in cell culture experiments.

### 2.3 Machine Learning Implementation

**FIGURE 4:**
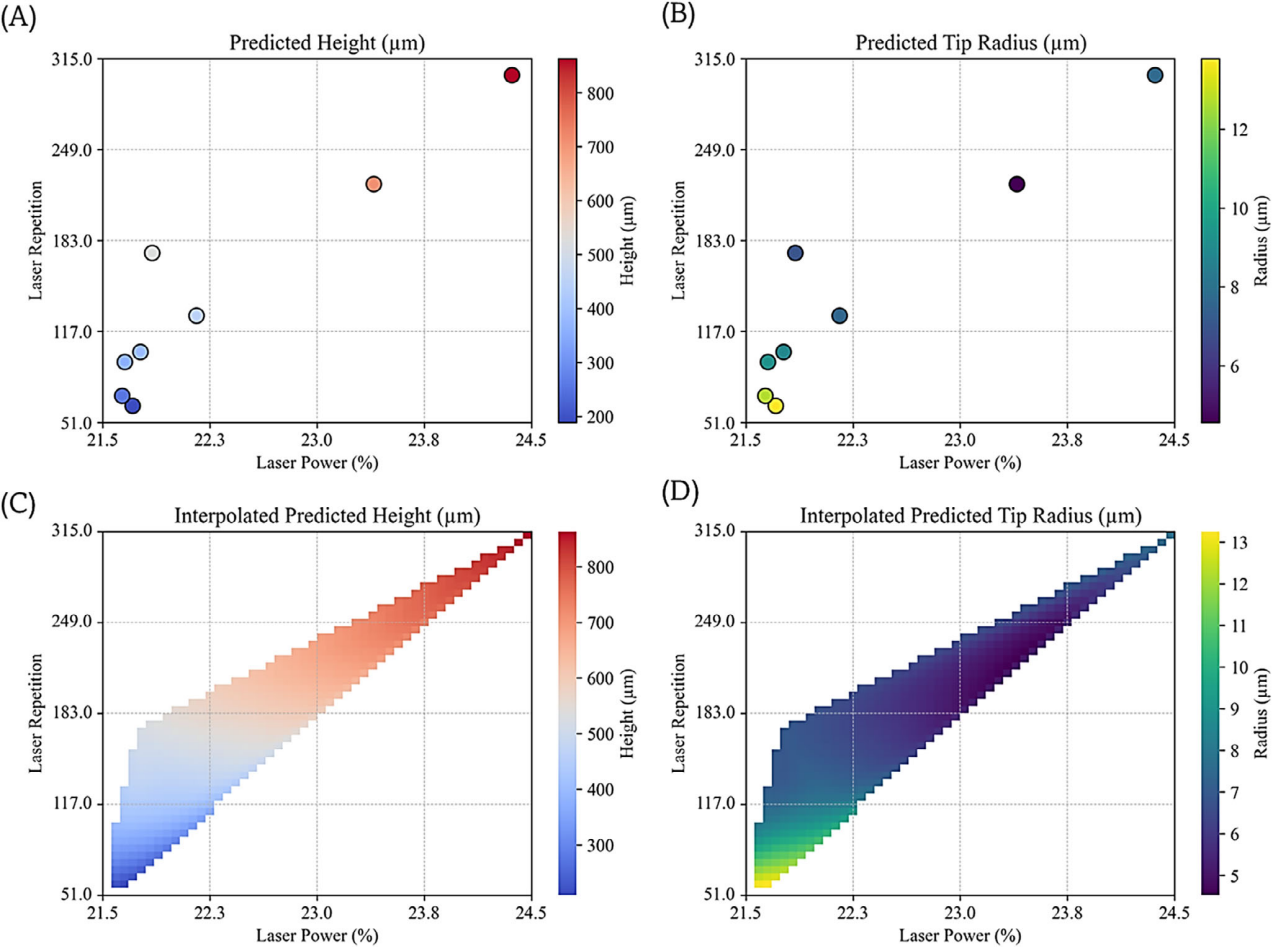
Effect of laser power and laser repetition on MN height and tip radius, along with interpolated predictions. (A) Scatter plot of predicted MN height as a function of laser power and laser repetition, with individual data points colored according to height values. (B) Scatter plot of predicted tip radius, where data points are colored based on tip radius values. (C) Heatmap of interpolated MN height predictions using cubic interpolation, demonstrating the trend of increasing height with higher laser power and repetition. (D) Interpolated heatmap of predicted tip radius, showing a decrease in tip radius as laser power and repetition increase. These results confirm the optimization trends observed in Table 2 and Figure 3, where an increase in laser parameters enhances MN height while reducing tip radius, consistent with the objective function described in equation 1.

The trained feedforward neural network models were evaluated based on their prediction accuracy for MN height and tip radius. Figure 4 presents scatter plots of predicted vs. actual values using the final trained weights, while Table 2 summarizes the MSE values across the training, test, and validation datasets. The results indicate strong generalization for both models, with the height model achieving higher accuracy, as evidenced by its lower MSE values. While the height model maintained consistent performance across all datasets, the tip radius model showed slightly reduced generalization, likely due to greater variability in tip radius measurements.

**TABLE 2:** Mean Squared Error (MSE) values for the trained feedforward neural network models predicting MN height and tip radius across training, test, and validation datasets. The height model exhibits lower MSE values, indicating higher accuracy and better generalization compared to the tip radius model.

Data Set	Height MSE	Tip Radius MSE
Training	0.0009	0.0071
Test	0.0012	0.0106
Validation	0.0007	0.0062

Following model training, the optimization process was conducted to determine the optimal input parameter sets for achieving predefined target heights while minimizing the tip radius. The optimal input parameters for each target height, along with the corresponding predicted MN characteristics, are presented in Table 3.

**TABLE 3:** Optimized laser processing parameters for achieving predefined MN heights while minimizing tip radius. The selected CAD size remained consistent at 50 µm, while laser power and repetition were adjusted to control MN height and tip sharpness.

Target Height (µm)	CAD Size (µm)	Laser Power (%)	Laser Repetition	Predicted Height (µm)	Predicted Tip Radius (µm)
190	50	21.61	51	188.80	13.79
249	50	21.53	59	254.22	12.74
359	50	21.55	86	357.52	9.28
382	50	21.67	94	381.55	8.88
448	50	22.10	123	449.02	7.62
524	50	21.76	173	523.57	7.00
712 865	50 50	23.46 24.52	228 315	711.07 863.35	4.56 7.73

As shown in Table 2, the optimization process resulted in a CAD size of 50 µm for each target height, which aligns with expectations. This outcome is supported by Figure 3, where a reduction in CAD size is associated with a smaller tip radius and an increase in height, consistent with the objective function outlined in Equation 1. Investigating the effects of laser power and laser repetition, Figure 4 displays a heatmap, with the *x*‐axis representing laser power and the y‐axis representing laser repetition. The individual data points for MN height and tip radius are shown in (A) and (B), while the cubic interpolation results are presented in (C) and (D). As illustrated, an increase in both laser power and laser repetition lead to a higher MN height, while simultaneously decreasing the tip radius.

#### 2.3.1 Evaluation of Prediction Power of Machine Learning Algorithm

In this study, feedforward neural networks (FNNs) were trained to predict the height and tip radius of MNs fabricated through laser processing of duralumin, with the aim of optimizing their design for specific transdermal applications. The models were trained on experimentally derived datasets linking laser parameters (power, CAD size, repetition rate) to MN morphologies, with input features normalized using min‐max scaling and optimized via Adam gradient descent (learning rate = 0.001, batch size = 16). Prior to model training, parameter combinations producing severely malformed or nonuniform structures were excluded from the dataset. These structures exhibited flattened or irregular tip morphologies that prevented reliable measurement of key geometrical descriptors such as tip radius and tip angle and did not satisfy the minimum structural requirements for functional skin‐penetrating microneedles. Therefore, the exclusion criteria were based on fabrication validity rather than model optimization. Nevertheless, this selection inherently constrains the predictive space of the current model to fabrication regimes capable of generating structurally functional MNs. We based our target MN height on literature values to ensure effective penetration of the epidermis (Table 4). This approach also helps avoid excessive depth, which could cause discomfort or tissue damage. By aligning our predictions with the known epidermal thicknesses for specific skin regions, we aimed to optimize the MN design for targeted applications such as vaccine delivery, insulin delivery, and therapeutic treatments for diabetic foot ulcers.

**TABLE 4:** The predicted and experimental MN heights for different skin parts were determined based on specific skin layer thicknesses. A uniform stratum corneum thickness of 15 µm was assumed for all skin types. The papillary dermis, the uppermost layer of the dermis, varies in thickness across skin regions; however, a standardized thickness of 100 µm was added to the total target thickness to ensure penetration into the upper dermis without damaging nerves. The total target thickness was calculated as the sum of the stratum corneum, epidermis, and 100 µm of the dermis. The MN height was obtained from three different batches produced with the same laser parameters to calculate the average MN height and ensure consistency and reproducibility.

Skin type	Common applications	Epidermis thickness (µm)	Dermis thickness (µm)	Target thickness (µm)	Predicted height (µm)	Experimental height (µm)	Predicted tip radius (µm)	Experimental tip radius (µm)	Optimal laser power (%)	Optimal laser repetition	References for skin thickness
Forearm (Dorsal)	Vaccine delivery	75	∼1350	190	188.77	168.8 ± 3.4	13.78	11.4 ± 1.9	21.61	51	[39]
Abdomen (Periumbilical region)	Insulin delivery	125	4550 ± 2147	249	248.46	207.9 ± 3.6	12.97	11.4 ± 0.4	22.71	58	[7]
Dorsum of foot (female)	Diabetic foot ulcer	267 ± 120	3184 ± 1273	382	381.54	341.9 ± 5.6	8.87	11.4 ± 1.5	21.67	94	[7]
Central forehead (male)	Wrinkle reduction	333 ± 21	1146 ± 100	448	449.03	439.4 ± 15.3	7.61	11.4 ± 1.8	22.09	123	[40]
Chin (female)	Acne scars	409 ± 27	1241 ± 68	524	523.71	517.3 ± 4.9	6.99	10.2 ± 0.7	21.75	173	[40]
Foot (plantar)	Targeting thick epidermis	596.6 ± 153	—	712	711.06	712.4 ± .7	4.55	10.2 ± 0.7	23.45	228	[6]
		749.3	—	865	865.34	890.3 ± 6.4	7.72	11.4 ± 0.4	24.51	315	

Model accuracy was evaluated by comparing predictions to experimental measurements, with performance quantified using mean absolute percentage error (MAE), root mean squared error (RMSE), and coefficient of determination (R^2^). Figure S5 shows one of the triplicate 3D microscope images, together with height, base diameter, tip angle, and tip radius, and the predicted values for different skin areas. This content directly supports the validation of the machine learning (ML) predictions, not the fabrication section or parameter screening. Based on the height predictions and actual height values provided in Table 4, the MAE, RMSE, and R^2^ were calculated as 20.36 µm, 25.00 µm, and 0.98, respectively. These results indicate a strong correlation between the predicted and actual height values, demonstrating the model's accuracy for height predictions. On the other hand, for the radius values, the MAE and RMSE were found to be 3.26 µm and 3.48 µm with R^2^<0, indicating a poor model fit. This suggests that improvements are necessary for the radius predictions, and exploring a different model architecture may be required to enhance the performance.

We observed that as the target thickness of the MNs decreases, the percentage error in predictions tends to increase. This can be attributed to the fact that at lower target heights, the MN tip geometry becomes less uniform due to challenges in controlling the laser processing parameters at such small scales. Additionally, the higher error at smaller thicknesses may also be due to inconsistencies in the available data, as smaller MNs are more sensitive to variations in processing conditions. To improve predictions, more consistent and comprehensive data at lower thickness ranges need to be obtained to better train the machine learning algorithm. Furthermore, incorporating smaller CAD design values (between 25 and 50 µm) into the study would provide deeper insights into MN formation at reduced heights, helping to better understand the geometric and processing challenges associated with fabricating MNs at these scales. This approach could enhance the model's ability to predict and optimize MN designs across a wider range of target thicknesses.

In contrast, the predictions for tip radius exhibited a higher average percentage error of 29.89%, suggesting that the model struggles to accurately predict this parameter. For example, the predicted tip radius of 13.78 µm deviated from the experimental value of 11.4 ± 1.9 µm, resulting in a percentage error of 20.88%. Similarly, a predicted tip radius of 4.55 µm differed significantly from the experimental value of 10.2 ± 0.7 µm, leading to a substantial percentage error of 55.39%. The higher error in tip radius predictions may be due to several factors. First, the tip radius is influenced by more complex and less predictable factors, such as the laser beam's focal spot size, material properties, and thermal effects during processing. Localized melting and material redeposition during near‐infrared laser ablation may additionally generate small irregularities at the MN tip region, making precise prediction of tip radius more challenging than MN height prediction. Unlike MN height, which mainly depends on cumulative vertical ablation depth, tip radius is highly sensitive to local thermal fluctuations, beam‐material interactions, and partial resolidification effects occurring at the microscale. Second, the experimental measurements of tip radius may be more susceptible to variability due to the challenges associated with accurately measuring such small features. Nevertheless, the experimentally obtained tip radius values remained within ranges previously reported in the literature for successful skin penetration [36]. Previous studies have demonstrated that MN insertion efficiency is strongly influenced by tip geometry and that decreasing tip radius reduces insertion force requirements and improves penetration performance. In the present study, the experimentally measured tip radii generally remained around 10–11 µm, which remained within ranges commonly used for successful transdermal insertion [37, 38]. To improve the accuracy of tip radius predictions, several strategies could be explored. Enhancing the machine learning model by incorporating additional parameters, such as laser beam characteristics and material thermal properties, may improve its predictive capability.

### 2.4 Photothermal Activity of the Fabricated MNs

**FIGURE 5:**
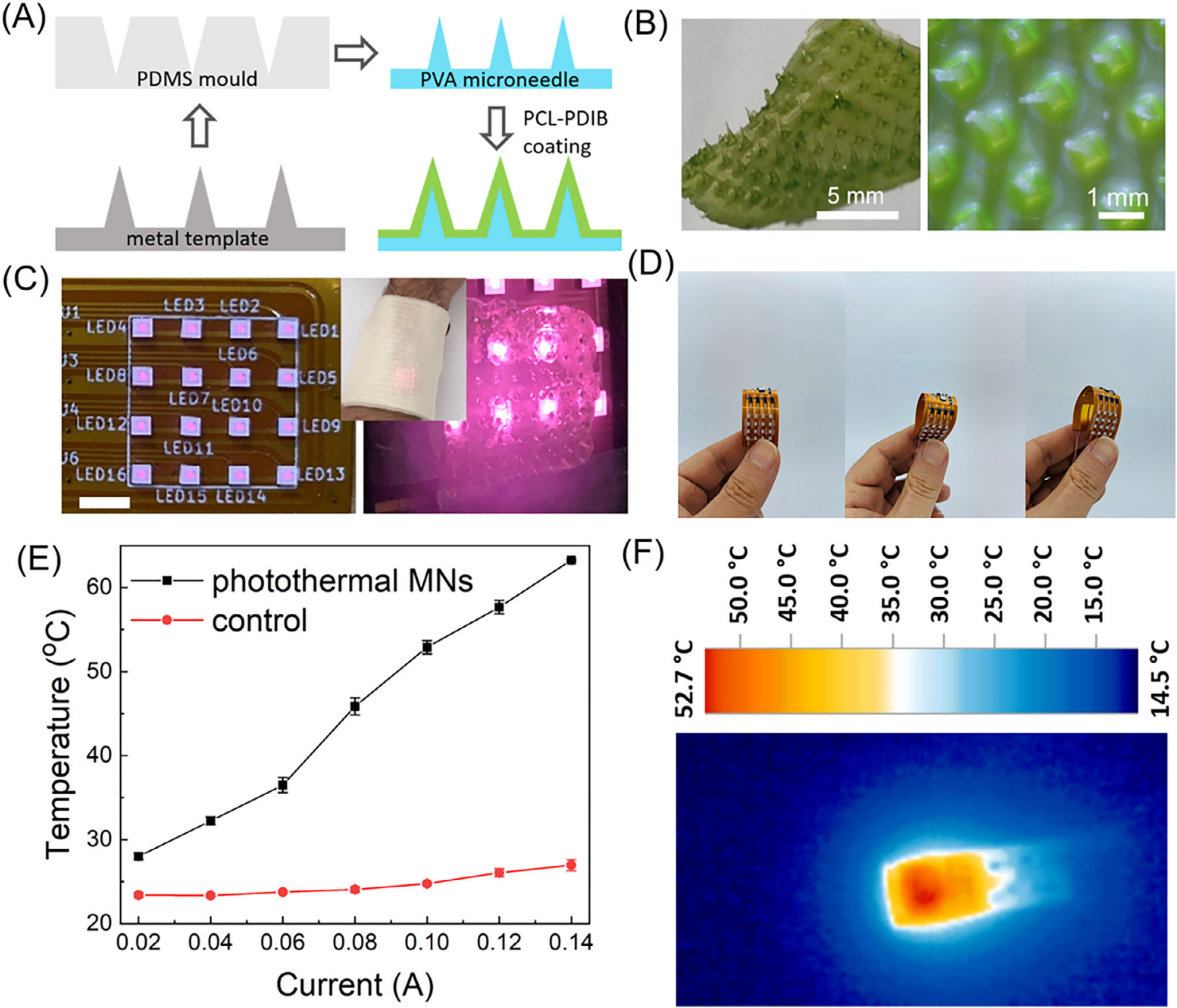
(A) Schematic illustration of the fabrication process for customized MNs designed for photothermal applications using machine learning‐assisted near‐infrared (NIR) laser processing, where the ML model selected both the target microneedle height (712 µm) and the optimized laser parameters used to fabricate the metal master mold. (B) Photograph (left) and optical microscopy image (right) of a photothermal PVA‐PCL MNs array. (C) Photograph of a flexible NIR LEDs chip made on polyimide substrate with 16 micro‐scale LEDs organized in a 4 × 4 square array, shown together with a photothermal MN patch mounted on an LED strip. The PVA/PCL MNs were molded from the ML‐optimized metal master via PDMS replication, ensuring that the final MNs inherit the precise height and tip sharpness required for consistent NIR‐driven photothermal activation. The inset picture shows the LED strip covered with a fabric bandage. The white scale bar is 2 mm. (D) Photograph demonstrating the mechanical flexibility and bendability of the fabricated photothermal MN substrate under manual deformation. (E) Temperature change of photothermal MNs and control MNs under illumination with a single LED for the current range from 20 mA to 140 mA. Temperature values represent the average of three repeated measurements (*n* = 3). Since the standard deviation values were very small (0.12–1.01°C) and difficult to visualize clearly in the graph, they are provided here in the caption for clarity. (F) Thermal camera image of MNs patch under illumination with LEDs strip powered with a battery. This photothermal demonstration is a direct downstream translation of the ML‐guided fabrication pipeline, showing how predictive geometry control enables functional device performance.

The MN height used for the photothermal array (712 µm) was selected using the ML optimization framework. This geometry corresponded to the optimized MN design targeting thick skin regions such as the plantar foot, based on literature‐reported skin thickness values summarized in Table 4. The ML‐derived laser parameters produced a high‐fidelity master template, enabling accurate PDMS replication and ensuring geometric fidelity in the final polymer MNs. The ML‐derived laser parameters produced a high‐fidelity master template, enabling accurate PDMS replication and ensuring geometric fidelity in the final polymer MNs. By feeding the ML‐optimized geometry into the molding process, we ensured that the PVA/PCL MNs maintained the precise tip sharpness and height required for effective NIR photothermal activation. Figure 5‐A schematically illustrates the fabrication process of the PVA‐PCL MNs using micromolding on PDMS female mold. PVA constitutes the core of the MNs, which were arranged into a 9×9 square array with an interspacing distance of 1.5 mm, and the outer PCL layer acts as photothermal film (Figure 5‐B, left image). Optical microscopy image shows that optimum PCL concentration of 2% resulted in a thin film covering the PVA MNs tips and the backing layer uniformly (Figure 5‐B, right image). Each MN has a base diameter of approximately 250 µm and a height of approximately 712 µm. The green color of the MNs array is sourced from the NIR dye doped in PCL. NIR dye was uniformly dispersed in the film owing to its very good solubility in PCL solution. The NIR dye shows a broad absorption peak around 800–1100 nm (Figure S6), which is within the safe wavelength range for applying on skin. Figure 5‐C illustrates the flexible NIR LED strip, in which microscale LEDs were arranged in a 4 × 4 array. Each LED chip (1.6 mm × 1.6 mm) was spaced 2 mm apart, covering a total area of approximately 15 mm × 15 mm. The LEDs emitted light at 860 nm (red), which was visible to the naked eye. High‐power LEDs were selected to provide sufficient excitation energy for the NIR dye. Figure 5‐D shows the bendability of the LED‐integrated substrate.

A microcontroller unit and LED drivers were integrated into the flexible circuit to enable independent switching control of the four LED rows. The circuit design also supports remote operation via Bluetooth using smart devices. The entire flexible LED strip and MN patch were encapsulated within a fabric bandage, forming a smart dressing suitable for application in chronic wound care and management (Figure 5‐C). We performed a compression test using a Mark‐10 F305 force gauge. The microneedles were subjected to applied forces of 0.5 N, 2 N, and 20 N to evaluate possible deformation or fracture of the microneedle tips. Optical microscopy images collected before and after compression show that the microneedles maintained their overall morphology after compression, indicating sufficient mechanical robustness under the tested loading conditions (Figure S7). The MNs array was also tested for its insertion to Parafilm M as an artificial skin simulant. Initially, no insertion was observed under 1 N applied force, partial insertion marks appeared at 3 N and successful penetration of the polymeric film was achieved at 5 N loading, confirming the mechanical penetration capability of the fabricated polymeric MNs under applied force (Figure S8).

Furthermore, a thermal camera was used to evaluate the photothermal temperature rise of the MNs. To evaluate the precise temperature rise within 25–65°C, a benchtop power supply was used, and MNs were illuminated with a single LED. The LED driver chip, CAT4104, enabled regulation of the LED‐drawn current and, consequently, the radiated optical power. The temperature was recorded for 2 min under the thermal camera, and the performance was compared to a control MN array that did not include the NIR dye. MNs showed a sensitive temperature increase up to 63°C with increasing current from 20 mA to 140 mA while plain MNs remained at 26°C (Figure 5‐E). Photothermal MNs LEDs also showed a higher heating rate than the control MNs (Figure S9). When all sixteen LEDs were powered with a rechargeable battery, MNs temperature increased to 52.7°C (Figure 5‐F). This temperature was measured around the central region of the MN array, while the surrounding regions were observed to be colder, as revealed in the thermal image with a color transition from red to yellow. This is due to the total surface of the LED array (1.5 cm × 1.5 cm), which is smaller than that of the MN array (2 cm × 2 cm), resulting in non‐total surface coverage of the MN array. Furthermore, low interspacing distance (2 mm) between adjacent LEDs was attributed to promoting heat accumulation in the central region. To optimize the system, interspacing distance can be increased, and the LED strip can be designed with an irradiation area larger than the microneedle array, allowing the entire microneedle surface to be covered and heated more uniformly. Furthermore, the measured temperature was lower than that obtained with a single LED, as shown in Figure 5‐E (63°C). The photothermal layer was a single layer here, whereas it was a double layer in single LED measurements, enabling stronger photothermal conversion. The results show that the irradiation intensity and resulting temperature can be precisely adjusted to maintain the microneedle temperature within a safe therapeutic range by adjusting the driving current. This bendable substrate delivers both comfort and performance needed for on‐body sensing and therapeutic applications. As a future work, a controlled release patch can be produced by simply encapsulating drugs in PVA‐PCL core‐shell structure, and a pulsatile release can be obtained by controlling the PCL melting via this smart photothermal heating.

## 3 Conclusion

Precision in MN geometry is essential for effective transdermal applications, as different skin regions require tailored structural properties to ensure optimal performance. Laser‐based fabrication offers significant advantages over other techniques and enables the precise control of MN geometry with high reproducibility. By optimizing laser power, repetition count, and CAD design parameters, we achieved highly specific MN geometries tailored for transdermal applications, considering variations in skin thickness and elasticity across different areas of the human body. Integrating machine learning further enhances this process by predicting optimal fabrication parameters, reducing trial‐and‐error experimentation, and ensuring consistent MN production with desired geometries. This predictive capability facilitates the efficient fabrication of MNs tailored for specific applications, improving scalability and customization for transdermal drug delivery and biomarker sensing. Future studies may further expand this approach by incorporating biological and biomechanical properties of different skin models, including skin thickness, elasticity, hydration state, insertion resistance, and anatomical variability across different species or body regions. Such integration could support the prediction of MN geometries better suited for specific tissue conditions and transdermal applications. For example, MNs intended for murine skin, human skin, or mechanically dynamic regions may require substantially different geometrical and mechanical characteristics to achieve reliable penetration while maintaining structural integrity and patient comfort. Combining tissue‐related parameters together with fabrication conditions may therefore improve the development of more application‐specific and biologically relevant MN designs.

Our results demonstrated that the machine learning model accurately predicted MN geometry, with minimal deviation from experimental values, ensuring reproducibility and reliability in fabrication. For instance, it can be adapted to fabricate ultra‐sharp MNs for delicate regions like the eyelids, where minimal invasiveness is required, or sturdier MNs for thicker skin areas such as the soles or palms, ensuring effective penetration and functionality. Furthermore, this laser‐guided approach extends beyond biomedical applications and can be adapted for use in agriculture and food processing. Beyond fabrication, the photothermal platform also opens opportunities for integrating controlled thermal dosing into wound care, antimicrobial treatments, and other localized therapies where precise geometry and predictable heating profiles are essential. The method was used to produce MNs assembled on a flexible NIR LEDs strip, which is promising for photothermal therapy applications. The photothermal system shows how ML‐optimized microneedle geometry can be directly translated into therapeutic devices. This result highlights that controlling geometry via ML‐guided laser processing is not only a fabrication advance but also a necessary step toward achieving consistent thermal performance in microLED‐driven photothermal therapies. While previous machine learning‐assisted laser studies have primarily focused on general process optimization, signal analysis, or manufacturing monitoring, the present work specifically demonstrates a predictive fabrication workflow linking ML‐guided NIR laser processing, experimentally validated MN fabrication, PDMS replication, and downstream wearable photothermal device integration for transdermal applications. Collectively, these results demonstrate the potential of data‐driven NIR laser processing for predictive and application‐specific MN fabrication while highlighting opportunities for future integration with therapeutic and biosensing platforms. This approach can be extended to other MN types and materials, further enhancing its applicability in transdermal drug delivery and sensing.

## 4 Experimental Section

### 4.1 Materials

Duralumin (200 mm × 300 mm × 1.2 mm thickness) metal sheet was obtained from RS Electronics (London, UK). Isopropanol (analytical grade, Sigma Aldrich, UK) was used for cleaning metal MNs and PDMS molds. Compressed air (BOC Gases, UK) was employed on the metal surface after the processing steps to remove any dust particles. MICRO 90 alkaline cleaner was obtained from Agar Scientific (UK) and used to facilitate the PDMS peeling‐off process.

### 4.2 Laser Processing

A 3‐axis Hybrid Laser Marker (MD‐X series, Keyence, Japan) equipped with a YVO_4_ laser operating at a wavelength of 1064 nm was used for processing Duralumin templates. The metal sheets were subjected to varying laser power settings ranging from 20% to 40% and laser repetition counting between 150 and 400 with a step of 50. The laser scanning speed was consistently maintained at 500 mm/s throughout the experiments. The effects of these parameters on critical MNs’ geometric features, including height, base diameter, aspect ratio, tip angle, and tip radius, were systematically analyzed and measured under the microscope. Additionally, the computer‐aided design (CAD) configuration was designed as a square shape, with side lengths of 25 µm, 50 µm, 75 µm, 100 µm, and 125 µm (Figure S10). The designs were drawn using Autodesk AutoCAD 2024 and saved as. dxf files. These files were processed using Marking Builder Plus (Keyence software) and hatched, then saved as. mlg files to be able to process with the laser. The metal plate was positioned on the translation stage, and the laser beam was precisely focused using software to ensure that the metal surface and the laser beam intersected accurately at the focal point. The focusing process was repeated each time a sample was removed from and repositioned on the translation stage. The production to obtain data for our prediction model was conducted with a 5 mm × 5 mm area to speed up the process, while the fabrication of MNs for casting and molding tests with PDMS was conducted as a larger area with a side length of 15 mm.

### 4.3 Morphological Characterization of Solid MNs

#### 4.3.1 Digital Microscopy Analysis

Preliminary visualization was assessed using light microscopy (GX Microscopes, GT Vision Ltd, UK). Digital microscopy was conducted using a Keyence VHX‐7000 digital microscope (Keyence, Japan) to evaluate the morphology of the metal MNs. The MNs were imaged at various magnifications to assess their geometric features, including height, base diameter, aspect ratio, tip angle, and tip diameter. The microscope's integrated software was utilized for precise measurements of these parameters to ensure accurate characterization of the MN structure. This analysis provided an initial qualitative and quantitative assessment of the MN fabrication quality.

#### 4.3.2 Scanning Electron Microscopy Analysis

Scanning electron microscopy (SEM) was performed using a Zeiss Sigma 300 SEM equipped with an Oxford Instruments Ultim Xtreme X‐ray detector at the Electron Microscopy Facility, Dunn School, University of Oxford. The Duralumin MN samples were sputter‐coated with a thin layer of palladium‐gold to enhance conductivity and prevent charging during imaging. The samples were mounted on SEM stubs with carbon tape and observed at an accelerating voltage of 20 kV. High‐resolution images were captured to analyze surface texture, tip integrity, and any microstructural defects or deformations resulting from the laser fabrication process.

#### 4.3.3 Surface Characterization with Profilometer

A 3D laser scanning microscope (VK‐X100, Keyence, Osaka, Japan) equipped with a 20–200× lens was utilized to assess the structural properties of the metal MNs. The same microscope was employed to investigate surface profile parameters, including roughness, skewness, and kurtosis, of the metal MNs. The analysis followed the JIS B 0601: 2001 (ISO 4287: 1997) standard, with λs and λc cut‐off values set at 0.25 µm and 0.8 mm, respectively.

### 4.4 Molding and Peeling Test with Polydimethylsiloxane (PDMS)

To assess the feasibility of using the fabricated Duralumin MNs as a template for PDMS molding, a series of casting tests was performed. Sylgard 184 (Dow Corning) PDMS elastomer was prepared by mixing the base and curing agent in a 10:1 ratio by weight. The mixture was thoroughly stirred and then degassed in a vacuum chamber for 30 min to remove air bubbles. The degassed PDMS was carefully poured over the Duralumin MN template, ensuring complete coverage of the MN structures. The PDMS‐coated template was then cured in an oven at 80°C for 2 h. After cooling to room temperature, the cured PDMS was carefully peeled off the Duralumin surface. The ease of removal and the quality of the resulting PDMS negative mold were assessed visually. This process was repeated multiple times to evaluate the reusability of the Duralumin template and the consistency of mold quality. The success of the PDMS peeling process provided insights into the surface characteristics of the Duralumin MNs and their suitability for creating reusable molds for subsequent polymeric MN fabrication.

### 4.5 Machine Learning Implementation

Two distinct feedforward ANNs with identical architectures were developed for distinct predictive tasks: (1) MN height estimation and (2) tip radius characterization. Both models incorporated laser power, CAD dimensions, and laser repetition frequency as input parameters. The training dataset was derived from experimental measurements, each conducted in triplicate under consistent input conditions. The experimental design encompassed CAD dimensions of 50, 75, and 100 µm; laser power settings ranging from 20% to 40% with 5% increments; and laser repetition frequencies from 50 to 400 with increments of 50.

Prior to model training, data preprocessing was done by first, eliminating parameter sets affected by manufacturing inconsistencies, which compromise MN quality. This step, alongside data cleaning, normalization, and outlier detection, helped eliminate unreliable data points that could impact predictive performance. Furthermore, the parameter ranges associated with manufacturing inconsistencies were excluded from the boundary constraints during the optimization process, ensuring that only high‐quality data informs the selection of input parameter sets for achieving the target heights. The heatmap of the input data, depicting the average of experimental measures, is illustrated in the results and discussion part (Figure 3) in which the white regions represent the previously indicated low‐quality MNs.

As illustrated in Figure S11‐A, the ANN consisted of six fully connected layers: an input layer with three neurons (corresponding to laser power, CAD size, and laser repetition), followed by four hidden layers with 8, 16, 32, and 8 neurons, and an output layer with a single neuron, representing either the height or the tip radius. Input features and target variables (height/tip radius) were normalized to a range using min‐max scaling to mitigate feature dominance. Each hidden layer incorporated layer normalization and the LeakyReLU activation function with a default alpha value of 0.3, enhancing model robustness by mitigating internal covariate shift and preventing vanishing gradients, thus facilitating stable training dynamics [41]. The models were trained using the Adam optimizer with a learning rate of 0.001 and the Mean Squared Error (MSE) loss function. Early stopping was applied with a patience of 100 epochs for the height model and 25 epochs for the tip radius model, ensuring that the best model weights were restored. The dataset was divided into 60% training, 20% validation, and 20% test sets for the height model, while the tip radius model was trained using 90% of the data, with 5% allocated for validation and 5% for testing. Training was performed with a batch size of 16 for up to 1000 epochs. Figure S11‐B presents the scatter plots comparing the predicted values against the actual values for the height and tip models using the training data and the test data. In this figure, the red dashed line represents the ideal 1:1 correlation (perfect prediction), and the blue dots indicate individual data points.

After training and obtaining the weights for both models, an optimization‐based approach was implemented to identify the optimal process parameters for achieving predefined MN target heights (190, 249, 359, 382, 448, 524, 712, and 865 µm while minimizing the tip radius. The optimization was formulated as a minimization problem, where the objective function consisted of terms for the height deviation from the target value and the corresponding tip radius. In detail, the objective function was defined as shown in equation 1.

(1) $$Z=w_{h}\left|H_{pred}-\;H_{target}\right|+w_{r}R_{pred}$$

where *H_pred_* and *R_pred_* are the predicted height and tip radius, while *H_target_* represents the target height. Similar to the neural network training, these values in the objective function were normalized using min‐max scaling and subsequently denormalized for further analysis. The weights were set as *w_h_* = 0.8 and *w_r_* = 0.2 to prioritize height accuracy while considering tip radius minimization. The optimization constraints included:

- Laser power between 20% and 40%.
- CAD size limited to 50, 75, or 100 µm.
- Laser repetition ranging from 50 to 400.
- Exclusion of parameter sets associated with low‐quality MNs.

During the optimization, a differential evolution (DE) algorithm was employed. Constraints were handled by rounding the CAD size to the nearest valid discrete value (50, 75, or 100 µm). Additionally, parameter sets that led to undesirable fabrication outcomes were penalized by assigning a large objective function value. The optimization was performed with a population size of 30, a maximum of 50 iterations, and a tolerance of 0.01 to ensure convergence. The trained feedforward neural network models for height and tip radius prediction were used to find the predicted values. The optimized parameter sets and their corresponding predicted MN characteristics were recorded for further validation.

To assess the predictive accuracy of the model for height and radius values, Mean Absolute Error (MAE) (equation 2), Root Mean Squared Error (RMSE) (equation 3), and the Coefficient of Determination (R^2^) (equation 4) are calculated using the formulas below, based on the values in Table 4.

(2) $$MAE=\frac{1}{n}\sum \left|y_{true}-y_{pred}\right|$$

(3) $$RMSE=\sqrt{\frac{1}{n}\sum {\left(y_{true}-y_{pred}\right)}^{2}}$$

(4) $$R^{2}=1-\frac{\sum {\left(y_{true}-y_{pred}\right)}^{2}}{\sum {\left(y_{true}-{\bar{y}}_{true}\right)}^{2}}$$

### 4.6 Photothermal MN Array

#### 4.6.1 NIR‐Responsive Dye Synthesis

N,N,N″,N″‐tetrakis(p‐diisobutylaminophenyl)‐p‐phenylenediimmonium bis(oxalato)borate was synthesized using the previously reported method [42]. Briefly, 1 g of a neutral amine compound N,N,N″,N″‐tetrakis(p‐diisobutylaminophenyl)‐p‐phenylenediamine was mixed with 500 mg of lithium bis(oxalate)borate. Then, 3.5 mL of dichloromethane and 2.5 mL of ethanol were added followed by stirring for 2 h. 330 mg of sodium persulfate and 6.7 mL of deionized water were added and stirred for further 2 h. Finally, 150 mL of dichloromethane and 200 mL of deionized water were added followed by vigorous shaking. Dichloromethane was separated from the aqueous phase by using a separatory funnel and completely evaporated by using a rotary evaporator, yielding a dark green powder.

#### 4.6.2 PVA MN Array Fabrication

PVA MNs were fabricated using a PDMS mold prepared from a MN template designed based on machine learning‐predicted parameters (712 µm in height). PDMS was prepared by mixing the base and curing agent at a 10:1 weight ratio, degassed to remove bubbles, and poured over the master template. The mixture was cured at 120°C to obtain a female mold. Separately, a 15% (w/w) PVA solution was prepared using 99% hydrolyzed PVA with an average molecular weight of 89,000–98,000 (Sigma‐Aldrich) in deionized water. The solution was stirred continuously and heated at 90°C overnight until fully dissolved. Approximately 500 µL of the prepared PVA solution was dispensed onto the mold, ensuring full coverage and infiltration into the dye‐loaded cavities, followed by centrifugation at 4000 rpm for 5 min. An additional 1 mL of PVA solution was added to form the MN backing layer, and the mold was placed in an oven at 40°C for 24 h. After drying, the solidified PVA MNs were carefully peeled off. In parallel, a solution of PCL in toluene was prepared with 10 % (w/w) concentration, and 15 mg of the NIR dye was added to it. The solution was diluted with toluene by 5‐fold, and the needle surface of the dried PVA array was dip‐coated with the diluted PCL solution. The array was left to dry in fumehood cupboard overnight. The overall fabrication workflow is illustrated in Figure S12, which outlines the laser machining of the master template, PDMS mold preparation, PVA casting, and subsequent dip‐coating of the photothermal layer

#### 4.6.3 Setup for microLED Integration to Illuminate Photothermal MNs

The wearable infrared (IR) module integrates sixteen high‐efficiency LEDs (OSRAM SFH series, 860 nm), each delivering a radiant power of approximately 1.15 W. The emitters are mounted on a flexible polyimide substrate coated with copper to provide mechanical flexibility and efficient thermal dissipation. The LEDs are arranged within a compact 15 mm × 15 mm footprint, generating a concentrated and spatially uniform illumination field suitable for near‐field optical applications.

Precise current regulation and stable optical output are achieved using CAT4104 LED driver integrated circuits (ICs), each capable of independently controlling four LEDs. In total, four CAT4104 drivers are employed to manage the sixteen LEDs, enabling fine adjustment of drive current and modulation of light intensity for experimental or therapeutic applications. Power for the LED array is supplied by a TPS62903 high‐efficiency buck converter, which steps down the system voltage to the regulated level required by the drivers while minimizing conversion losses and thermal buildup.

The entire system is controlled by a Nordic nRF52811 Bluetooth Low Energy (BLE) microcontroller, which provides wireless communication and programmable control of illumination patterns. The nRF52811's low‐power operation enhances energy efficiency, extending the module's operational lifetime in wearable applications. The circuit is powered by an external rechargeable battery, interfaced through the power management stage incorporating the TPS62903 converter and associated filtering components to ensure stable, noise‐free operation.

#### 4.6.4 Thermal Characterization of NIR‐Induced Heating in PVA MNs

We used a thermal camera (testo 875‐1) for the recording of the temperature of MNs‐flexible LEDs strip. The camera was held at a distance of 10 cm from the MNs surface. The MNs were located on a flexible LED strip at a 2 mm distance using a 3D‐printed support, and their needle surface was facing up. The thermal camera was calibrated with an emissivity value of 0.94 and the reflected temperature compensation (ambient temperature) as 21°C.

### 4.7 Statistical Analysis

Statistical analyses were conducted using GraphPad Prism to evaluate the effects of laser processing parameters on MN features, including height, base diameter, tip angle, curvature radius, and aspect ratio. For this purpose, three MNs were randomly selected from each experimental condition after laser processing to create the dataset. A one‐way ANOVA test was used to compare the effects of laser power, repetition, and CAD design size on the MN features (Supplementary material S13). Graphical representations, including bar charts with error bars, were generated to visualize the differences between groups. Statistical significance was determined with a *p*‐value threshold of <0.05.

## Conflicts of Interest

The authors declare no conflict of interest.

## Supporting information




**Supporting File**: smll74545‐sup‐0001‐SuppMat.docx.

## Data Availability

The data that support the findings of this study are available in the supplementary material of this article.
