# Supplementary material for: Machine Learning‐Guided Near‐Infrared Laser Processing for Precise Geometry Control in Microneedles with Downstream microLED‐Triggered Photothermal Functionality
Source: Small. 2026 Jul 16;22(48):e74545. doi: 10.1002/smll.74545 (PMC13509086; doi:10.1002/smll.74545)
Supplement: Supplementary file 1 [file SMLL-22-e74545-s001.docx]

**SUPPLEMENTARY MATERIALS**

**Machine learning-guided near-infrared laser processing for precise geometry control in microneedles** **with downstream microLED-triggered photothermal functionality**

Ismail Eş^1^, Burak M, Gormus^2^, Pinar Ayas^3^, Irfan Ullah^3^, Christopher M. Proctor^1*^

^1^ Institute of Biomedical Engineering, Old Road Campus Research Building, University of Oxford, Oxford, United Kingdom

^2^ Department of Microsystems Engineering, University of Freiburg, Georges-Koehler-Allee 101, 79110 Freiburg, Germany

^3^ School of Electronics and Computer Science, University of Southampton, University Road, Southampton, SO17 1BJ United Kingdom

**^*^Corresponding author:**

Christopher M. Proctor (christopher.proctor@eng.ox.ac.uk)

**Figure S1.** Microscope images of malformed MN tips at specific laser parameters

**Figure S2**. The effect of laser parameters (CAD size, laser power, and laser repetition) on critical MN features including height, base diameter, aspect ratio, tip angle, and tip radius. ANOVA analysis confirmed that Size of CAD Design, Laser Power, Laser Repetition, and their interaction terms significantly affected the microneedle geometrical responses (p < 0.001).

**Figure S3.** 3D surface topography analysis of duralumin metal processed with varying laser power values at a fixed laser repetition of 50, captured using a Keyence digital microscope. Each image represents the surface morphology and height variations induced by different laser power settings and shows the progressive changes in roughness and texture as the power increases. The color scale denotes the surface height variations, with blue indicating lower regions and red representing higher elevations.

**Figure S4.** Comparison of the height profiles of the master template and the corresponding PDMS mold. (Left) 3D surface plot, heatmap, and height profile of the MN structure on the duralumin master template after laser processing. (Right) 3D surface plot, heatmap, and height profile of the PDMS mold replicated from the master template. The height measurements indicate a precise transfer of the MN geometry from the master template to the PDMS mold. The replication fidelity, which represents how accurately the PDMS reproduced the original metal MN geometry, was calculated to be approximately 99.93%, while the percentage deviation between the master and replicated structure was only 0.065%.

**Figures S5**. The 3D microscope images and heat map of metal MNs showing MN height, base diameter, tip angle, and tip radius. Please note that one of the triplicates was shown in these images. The values for the skin areas predicted by machine learning algorithm are shown in the bottom right. The table shows the experimental MN geometrical measurements obtained from triplicate fabrication and characterization experiments used for validation of the machine learning predictions.

**Figure S6**. UV-Vis-NIR absorbance spectra of PDIB dye in toluene at increasing concentration that clearly shows broad absorption in the near-infrared region. The spectra exhibit a characteristic absorbance peak centered around 950 nm, confirming the dye’s suitability for photothermal activation under NIR light. Increasing dye concentration results in a proportional increase in absorbance intensity, indicating stable dispersion and strong NIR absorption properties critical for efficient light-to-heat conversion in MN-based applications.

**Figure S7**. Optical microscopy images of the MNs array (A) before and after undergoing a compression test under the pressures of (B) 1.54 kPa (0.5 N load), (C) 6.17 kPa (2 N load), and (D) 61.72 kPa (20 N load).

**Figure S8**. (A-C) Photographs of the setup prepared for the insertion test. Parafilm M was first overspread on a metal square frame, and the MN array was placed on it. Then the frame was placed under the load in compression tester machine (Mark-10 F305). (D) Optical microscopy image of the Parafilm M showing that the needles penetrated the Parafilm M after applying 5 N force (corresponding to an applied pressure of 15.43 kPa). Sites in the red dashed circles are the insertion marks.

**Figure S9**. Heating rate curve of the photothermal MNs and the control MNs under heating with sixteen NIR LEDs array on the flexible strip. Control sample is heated up to 38 ^ο^C due to the heat dissipation by the LEDs.

**Figure S10**. Schematic illustration of the microneedle array layout showing the center-to-center interspacing distance of 1.2 mm between adjacent microneedles. The magnified region highlights the local laser processing strategy, including the hatched and non-hatched regions used during fabrication. Preliminary experiments additionally confirmed that reducing the interspacing distance down to 0.6 mm did not produce significant differences in individual microneedle formation or geometry. This behavior was attributed to the selected spacing remaining compatible with the effective laser spot dimensions and avoiding excessive overlap between adjacent laser processing regions.

**Figure S11.** **(A)** Schematic representation of the neural network architecture used for predicting MN height and tip radius. The network consists of an input layer with three nodes corresponding to laser power, CAD size, and laser repetition, followed by four hidden layers containing 8, 16, 32, and 8 neurons, respectively. The output layer consists of a single neuron representing either MN height or tip radius. Each hidden layer incorporates layer normalization and the LeakyReLU activation function. The model is trained using the Adam optimizer with a learning rate of 0.001 and the Mean Squared Error (MSE) loss function, with early stopping applied to prevent overfitting. **(B)** Scatter plots illustrating the performance of the trained models for MN height and tip radius predictions. The predicted values are plotted against the actual values for both training and test datasets. The red dashed line represents the ideal 1:1 correlation, indicating perfect prediction, while the blue dots represent individual data points. The height model exhibits strong agreement with experimental data in both training and test sets, while the tip radius model shows greater variability, particularly for larger tip radii.

**Figure S12.** **ML-guided fabrication workflow for producing photothermal PVA MN arrays.** The process begins with the laser machining of a metal master template (1), where the MN geometry—predicted by the machine-learning model (712 µm height)—is transferred onto the substrate using optimized NIR laser parameters to generate a high-fidelity template. In the second stage (2), a PDMS female mold is prepared by mixing Sylgard 184 base and curing agent (10:1 w/w), followed by degassing, casting onto the laser-machined template, and thermal curing before peeling to obtain a precise negative mold. In the final stage (3), a 15% PVA solution is cast into the PDMS mold, degassed or centrifuged to ensure complete cavity filling, and dried to yield solid PVA MNs that replicate the ML-optimized geometry. The dried arrays are subsequently dip-coated in a diluted PCL/toluene solution containing the synthesized NIR dye, forming a uniform photothermal layer on the MN surfaces and completing fabrication of the photothermal PVA MN device.

**S13. Statistical analysis**

**General Linear Model: MN Height versus Size of CAD Design (μm), Laser POWER (%), Laser Repetition**

**Factor Information**

| **Factor** | **Type** | **Levels** | **Values** |
| --- | --- | --- | --- |
| Size of CAD Design  (um) | Fixed | 3 | 50, 75, 100 |
| Laser POWER (%) | Fixed | 5 | 20, 25, 30, 35, 40 |
| Laser Repetition | Fixed | 8 | 50, 100, 150, 200, 250, 300, 350, 400 |

**Analysis of Variance**

| **Source** | **DF** | **Seq SS** | | | **Contribution** | | |
| --- | --- | --- | --- | --- | --- | --- | --- |
| Size of CAD Design (um) | 2 | 5277233 | | | 24.32% | | |
| Laser POWER (%) | 4 | 1952842 | | | 9.00% | | |
| Laser Repetition | 7 | 12374814 | | | 57.02% | | |
| Size of CAD Design (um)*Laser POWER (%) | 8 | 462246 | | | 2.13% | | |
| Size of CAD Design (um)*Laser Repetition | 14 | 1092687 | | | 5.04% | | |
| Laser POWER (%)*Laser Repetition | 28 | 184335 | | | 0.85% | | |
| Size of CAD Design (um)*Laser POWER (%)*Laser Repetition | 56 | 170068 | | | 0.78% | | |
| Error | 240 | 186491 | | | 0.86% | | |
| Total | 359 | 21700717 | | | 100.00% | | |
| **Source** | **Adj SS** | | **Adj MS** | | | **F-Value** |  |
| Size of CAD Design  (um) | 5277233 | | 2638616 | | | 3395.70 |  |
| Laser POWER (%) | 1952842 | | 488211 | | | 628.29 |  |
| Laser Repetition | 12374814 | | 1767831 | | | 2275.06 |  |
| Size of CAD Design  (um)*Laser POWER (%) | 462246 | | 57781 | | | 74.36 |  |
| Size of CAD Design  (um)*Laser Repetition | 1092687 | | 78049 | | | 100.44 |  |
| Laser POWER (%)*Laser Repetition | 184335 | | 6583 | | | 8.47 |  |
| Size of CAD Design  (um)*Laser POWER (%)*Laser Repetition | 170068 | | 3037 | | | 3.91 |  |
| Error | 186491 | | 777 | | |  |  |
| Total |  | |  | | |  |  |
| **Source** | **P-Value** | | |  |  |  |  |
| Size of CAD Design  (um) | 0.000 | | |  |  |  |  |
| Laser POWER (%) | 0.000 | | |  |  |  |  |
| Laser Repetition | 0.000 | | |  |  |  |  |
| Size of CAD Design  (um)*Laser POWER (%) | 0.000 | | |  |  |  |  |
| Size of CAD Design  (um)*Laser Repetition | 0.000 | | |  |  |  |  |
| Laser POWER (%)*Laser Repetition | 0.000 | | |  |  |  |  |
| Size of CAD Design  (um)*Laser POWER (%)*Laser Repetition | 0.000 | | |  |  |  |  |
| Error |  | | |  |  |  |  |
| Total |  | | |  |  |  |  |

**General Linear Model: MN Base Diameter versus Size of CAD Design (um), Laser POWER (%), Laser Repetition**

**Analysis of Variance**

| **Source** | **DF** | **Seq SS** | | | **Contribution** | | |
| --- | --- | --- | --- | --- | --- | --- | --- |
| Size of CAD Design  (um) | 2 | 796396 | | | 21.72% | | |
| Laser POWER (%) | 4 | 137756 | | | 3.76% | | |
| Laser Repetition | 7 | 1292604 | | | 35.26% | | |
| Size of CAD Design  (um)*Laser POWER (%) | 8 | 539686 | | | 14.72% | | |
| Size of CAD Design  (um)*Laser Repetition | 14 | 545002 | | | 14.87% | | |
| Laser POWER (%)*Laser Repetition | 28 | 85043 | | | 2.32% | | |
| Size of CAD Design  (um)*Laser POWER (%)*Laser Repetition | 56 | 210883 | | | 5.75% | | |
| Error | 240 | 58706 | | | 1.60% | | |
| Total | 359 | 3666076 | | | 100.00% | | |
| **Source** | **Adj SS** | | **Adj MS** | | | **F-Value** |  |
| Size of CAD Design  (um) | 796396 | | 398198 | | | 1627.91 |  |
| Laser POWER (%) | 137756 | | 34439 | | | 140.79 |  |
| Laser Repetition | 1292604 | | 184658 | | | 754.92 |  |
| Size of CAD Design  (um)*Laser POWER (%) | 539686 | | 67461 | | | 275.79 |  |
| Size of CAD Design  (um)*Laser Repetition | 545002 | | 38929 | | | 159.15 |  |
| Laser POWER (%)*Laser Repetition | 85043 | | 3037 | | | 12.42 |  |
| Size of CAD Design  (um)*Laser POWER (%)*Laser Repetition | 210883 | | 3766 | | | 15.40 |  |
| Error | 58706 | | 245 | | |  |  |
| Total |  | |  | | |  |  |
| **Source** | **P-Value** | | |  |  |  |  |
| Size of CAD Design  (um) | 0.000 | | |  |  |  |  |
| Laser POWER (%) | 0.000 | | |  |  |  |  |
| Laser Repetition | 0.000 | | |  |  |  |  |
| Size of CAD Design  (um)*Laser POWER (%) | 0.000 | | |  |  |  |  |
| Size of CAD Design  (um)*Laser Repetition | 0.000 | | |  |  |  |  |
| Laser POWER (%)*Laser Repetition | 0.000 | | |  |  |  |  |
| Size of CAD Design  (um)*Laser POWER (%)*Laser Repetition | 0.000 | | |  |  |  |  |
| Error |  | | |  |  |  |  |
| Total |  | | |  |  |  |  |

**General Linear Model: Aspect Ratio versus Size of CAD Design (um), Laser POWER (%), Laser Repetition**

**Analysis of Variance**

| **Source** | **DF** | **Seq SS** | | | **Contribution** | | |
| --- | --- | --- | --- | --- | --- | --- | --- |
| Size of CAD Design  (um) | 2 | 2.411 | | | 3.37% | | |
| Laser POWER (%) | 4 | 11.546 | | | 16.12% | | |
| Laser Repetition | 7 | 36.632 | | | 51.15% | | |
| Size of CAD Design  (um)*Laser POWER (%) | 8 | 4.671 | | | 6.52% | | |
| Size of CAD Design  (um)*Laser Repetition | 14 | 4.092 | | | 5.71% | | |
| Laser POWER (%)*Laser Repetition | 28 | 2.434 | | | 3.40% | | |
| Size of CAD Design  (um)*Laser POWER (%)*Laser Repetition | 56 | 6.475 | | | 9.04% | | |
| Error | 240 | 3.364 | | | 4.70% | | |
| Total | 359 | 71.625 | | | 100.00% | | |
| **Source** | **Adj SS** | | **Adj MS** | | | **F-Value** |  |
| Size of CAD Design  (um) | 2.411 | | 1.20546 | | | 86.01 |  |
| Laser POWER (%) | 11.546 | | 2.88642 | | | 205.94 |  |
| Laser Repetition | 36.632 | | 5.23321 | | | 373.37 |  |
| Size of CAD Design  (um)*Laser POWER (%) | 4.671 | | 0.58382 | | | 41.65 |  |
| Size of CAD Design  (um)*Laser Repetition | 4.092 | | 0.29227 | | | 20.85 |  |
| Laser POWER (%)*Laser Repetition | 2.434 | | 0.08693 | | | 6.20 |  |
| Size of CAD Design  (um)*Laser POWER (%)*Laser Repetition | 6.475 | | 0.11563 | | | 8.25 |  |
| Error | 3.364 | | 0.01402 | | |  |  |
| Total |  | |  | | |  |  |
| **Source** | **P-Value** | | |  |  |  |  |
| Size of CAD Design  (um) | 0.000 | | |  |  |  |  |
| Laser POWER (%) | 0.000 | | |  |  |  |  |
| Laser Repetition | 0.000 | | |  |  |  |  |
| Size of CAD Design  (um)*Laser POWER (%) | 0.000 | | |  |  |  |  |
| Size of CAD Design  (um)*Laser Repetition | 0.000 | | |  |  |  |  |
| Laser POWER (%)*Laser Repetition | 0.000 | | |  |  |  |  |
| Size of CAD Design  (um)*Laser POWER (%)*Laser Repetition | 0.000 | | |  |  |  |  |
| Error |  | | |  |  |  |  |
| Total |  | | |  |  |  |  |

**General Linear Model: Tip Angle versus Size of CAD Design (um), Laser POWER (%), Laser Repetition**

**Analysis of Variance**

| **Source** | **DF** | **Seq SS** | | | **Contribution** | | |
| --- | --- | --- | --- | --- | --- | --- | --- |
| Size of CAD Design  (um) | 2 | 186.1 | | | 1.16% | | |
| Laser POWER (%) | 4 | 7311.2 | | | 45.71% | | |
| Laser Repetition | 7 | 3038.9 | | | 19.00% | | |
| Size of CAD Design  (um)*Laser POWER (%) | 8 | 821.2 | | | 5.13% | | |
| Size of CAD Design  (um)*Laser Repetition | 14 | 772.3 | | | 4.83% | | |
| Laser POWER (%)*Laser Repetition | 28 | 1299.1 | | | 8.12% | | |
| Size of CAD Design  (um)*Laser POWER (%)*Laser Repetition | 56 | 925.1 | | | 5.78% | | |
| Error | 240 | 1639.4 | | | 10.25% | | |
| Total | 359 | 15993.4 | | | 100.00% | | |
| **Source** | **Adj SS** | | **Adj MS** | | | **F-Value** |  |
| Size of CAD Design  (um) | 186.1 | | 93.04 | | | 13.62 |  |
| Laser POWER (%) | 7311.2 | | 1827.80 | | | 267.58 |  |
| Laser Repetition | 3038.9 | | 434.13 | | | 63.55 |  |
| Size of CAD Design  (um)*Laser POWER (%) | 821.2 | | 102.65 | | | 15.03 |  |
| Size of CAD Design  (um)*Laser Repetition | 772.3 | | 55.17 | | | 8.08 |  |
| Laser POWER (%)*Laser Repetition | 1299.1 | | 46.40 | | | 6.79 |  |
| Size of CAD Design  (um)*Laser POWER (%)*Laser Repetition | 925.1 | | 16.52 | | | 2.42 |  |
| Error | 1639.4 | | 6.83 | | |  |  |
| Total |  | |  | | |  |  |
| **Source** | **P-Value** | | |  |  |  |  |
| Size of CAD Design  (um) | 0.000 | | |  |  |  |  |
| Laser POWER (%) | 0.000 | | |  |  |  |  |
| Laser Repetition | 0.000 | | |  |  |  |  |
| Size of CAD Design  (um)*Laser POWER (%) | 0.000 | | |  |  |  |  |
| Size of CAD Design  (um)*Laser Repetition | 0.000 | | |  |  |  |  |
| Laser POWER (%)*Laser Repetition | 0.000 | | |  |  |  |  |
| Size of CAD Design  (um)*Laser POWER (%)*Laser Repetition | 0.000 | | |  |  |  |  |
| Error |  | | |  |  |  |  |
| Total |  | | |  |  |  |  |

**General Linear Model: Tip Radius versus Size of CAD Design (um), Laser POWER (%), Laser Repetition**

**Analysis of Variance**

| **Source** | **DF** | **Seq SS** | | | **Contribution** | | |
| --- | --- | --- | --- | --- | --- | --- | --- |
| Size of CAD Design  (um) | 2 | 60481 | | | 62.54% | | |
| Laser POWER (%) | 4 | 1693 | | | 1.75% | | |
| Laser Repetition | 7 | 6974 | | | 7.21% | | |
| Size of CAD Design  (um)*Laser POWER (%) | 8 | 2217 | | | 2.29% | | |
| Size of CAD Design  (um)*Laser Repetition | 14 | 4884 | | | 5.05% | | |
| Laser POWER (%)*Laser Repetition | 28 | 4357 | | | 4.51% | | |
| Size of CAD Design  (um)*Laser POWER (%)*Laser Repetition | 56 | 7539 | | | 7.80% | | |
| Error | 240 | 8565 | | | 8.86% | | |
| Total | 359 | 96709 | | | 100.00% | | |
| **Source** | **Adj SS** | | **Adj MS** | | | **F-Value** |  |
| Size of CAD Design  (um) | 60481 | | 30240.3 | | | 847.39 |  |
| Laser POWER (%) | 1693 | | 423.2 | | | 11.86 |  |
| Laser Repetition | 6974 | | 996.2 | | | 27.92 |  |
| Size of CAD Design  (um)*Laser POWER (%) | 2217 | | 277.1 | | | 7.77 |  |
| Size of CAD Design  (um)*Laser Repetition | 4884 | | 348.9 | | | 9.78 |  |
| Laser POWER (%)*Laser Repetition | 4357 | | 155.6 | | | 4.36 |  |
| Size of CAD Design  (um)*Laser POWER (%)*Laser Repetition | 7539 | | 134.6 | | | 3.77 |  |
| Error | 8565 | | 35.7 | | |  |  |
| Total |  | |  | | |  |  |
| **Source** | **P-Value** | | |  |  |  |  |
| Size of CAD Design  (um) | 0.000 | | |  |  |  |  |
| Laser POWER (%) | 0.000 | | |  |  |  |  |
| Laser Repetition | 0.000 | | |  |  |  |  |
| Size of CAD Design  (um)*Laser POWER (%) | 0.000 | | |  |  |  |  |
| Size of CAD Design  (um)*Laser Repetition | 0.000 | | |  |  |  |  |
| Laser POWER (%)*Laser Repetition | 0.000 | | |  |  |  |  |
| Size of CAD Design  (um)*Laser POWER (%)*Laser Repetition | 0.000 | | |  |  |  |  |
| Error |  | | |  |  |  |  |
| Total |  | | |  |  |  |  |

**ANOVA Results and Factor Contributions**

**MN Height:** All three factors demonstrated statistical significance at p < 0.001. Laser Repetition emerged as the most influential parameter, accounting for 57% of the total variation in microneedle height. Size of CAD Design and Laser Power contributed 24% and 9% of the variation, respectively. The model yielded an adjusted R² of 90%, indicating a strong explanatory capacity of the selected factors. A positive relationship was observed between Laser Repetition and microneedle height, whereby increasing repetition values corresponded to a progressive increase in height.

**MN Base Diameter:** All three factors were found to be statistically significant (p < 0.001). Laser Repetition was identified as the primary determinant of base diameter, explaining 35% of the total variation, followed by Size of CAD Design at 22%. Laser Power demonstrated the least contribution, accounting for approximately 4% of the variation. This outcome is consistent with the thermal accumulation hypothesis, wherein an increase in laser repetition amplifies the thermal effect on the substrate material, resulting in a lateral expansion of the base diameter.

**Aspect Ratio:** Laser Repetition remained the dominant factor for aspect ratio, contributing 51% of the total variation. Laser Power exhibited a comparatively greater influence on this response variable (16%) relative to its contribution to individual dimensional parameters, which can be attributed to the differential rate at which power affects height and diameter, thereby modulating the resulting aspect ratio. The contribution of Size of CAD Design was comparatively limited at 3.4%.

**MN Tip Angle:** A notable shift in factor significance was observed for microneedle tip angle. Laser Power emerged as the primary determinant, accounting for 46% of the total variation, followed by Laser Repetition at 19%. Size of CAD Design contributed minimally, explaining only 1.2% of the variation. These findings suggest that tip angle geometry is predominantly governed by laser power, likely due to the formation of a deeper and more geometrically constrained ablation profile at higher power levels.

**MN Tip Radius:** The analysis of microneedle tip radius revealed a distinctly different pattern of factor contributions compared to the other response variables. Size of CAD Design accounted for 62.5% of the total variation, indicating that tip sharpness is primarily determined by the geometric parameters of the CAD design rather than by laser processing conditions. Laser Repetition and Laser Power contributed 7.2% and 1.8%, respectively, suggesting that their influence on tip radius is statistically limited.
